# Shear capacity of tapered web panel of plate girders with web opening

**DOI:** 10.1038/s41598-026-67009-8

**Published:** 2026-08-23

**Authors:** Mohamed A. El Aghoury, Mohammed Taher, Sherif M. Ibrahim, Ahmed M. Ebid

**Affiliations:** 1 Faculty of Engineering, Ain Shams University, Cairo, 11535 Egypt; 2 Faculty of Engineering & Technology, Future University in Egypt, Cairo, 11865 Egypt

**Keywords:** Tapered plate girder, Slender web, Web opening, Shear capacity, Web plate buckling, Finite element modelling, Engineering, Materials science, Mathematics and computing

## Abstract

Tapered plate girders with web openings are widely used to route services and reduce weight, yet current provisions only partly capture the combined effects of tapering angle, opening size, panel aspect ratio, and web slenderness. A total of 115 shell finite-element (FE) models were analysed. The FE model was verified against measurements from seven tested tapered girders with web openings and then used in a full-factorial parametric study augmented to assess opening-shape effects. The verified model reproduced the experimental response and shows consistent trends. Capacity falls as the panel aspect ratio, web opening ratio and web slenderness ratio increase, while the tapering angle showed a beneficial influence within the investigated range, improving the web shear capacity in the presence of web openings. Based on the numerical dataset, an EC3-aligned mechanics-based equation was developed for direct shear-capacity prediction. The equation incorporates web slenderness, opening ratio, panel aspect ratio, and tapering angle, and its coefficients were obtained using nonlinear regression of the FE database. The calibrated model provides accurate predictions with good agreement with the FE results. Overall, the work links experiment, simulation, and design to give a more reliable basis for sizing tapered girders with web openings.

## Introduction

Tapered plate girders are widely used because varying depth along the span aligns material with demand, improving stress distribution and reducing both weight and cost. Openings in the web are routinely introduced to pass services, so their influence on shear capacity, bending resistance, and local buckling must be clearly considered. This is especially important in tapered girders, where the change in depth affects the stress flow within the web. In practice, tapered girders with web openings can provide safe and economical solutions. However, the combined effect of tapering and web openings should be directly considered, instead of relying on simplified rules developed for prismatic girders.

Recent studies have investigated this topic from different perspectives. Trahair and Ansourian^1^ examined the shear-stress fields in tapered I-beams and showed that standard beam theory may misrepresent the actual shear-flow pattern. A more accurate representation was obtained by modelling the stress path as radial stress trajectories. Using nonlinear finite elements, Serror et al.^2^ investigated tapered web panels by varying the main geometric parameters. The study showed that existing design rules may not fully capture stress redistribution and post-buckling reserve. Based on this, simple correction factors were proposed to improve the design predictions. Through testing and modelling, Tankova et al.^3^ explained the behaviour of non-prismatic columns, beams, and beam-columns when initial imperfections are considered. For lateral–torsional stability, Kucukler and Gardner^4^ introduced a stiffness-reduction method for welded web-tapered beams. The method considered plasticity and initial imperfections, and was validated using numerical models. Chockalingam et al.^5^ developed a mechanics-based equation to describe shear flow in tapered I-beams. The equation was verified using finite element results. For slender tapered members, Ibrahim et al.^6^ proposed modified shear-buckling coefficients; related axial work^7^ calibrated buckling coefficients for tapered webs in compression. At frame level, S. Ibrahim^8^ gave closed-form stiffness relations and effective length charts for tapered columns that indicated where the alignment charts fall short. At the detail and system scales, Saleh Amin et al.^9^ tested and modelled, under cyclic loading, beam–column connections with web openings. The results showed that the connection strength decreased when the openings became larger or were placed closer to the column face. The reduction was more significant when the openings were not reinforced. Optimisation by M. El Aghoury et al.^10^, combining Generalised Reduced Gradient (GRG) with Nonlinear Regression (NLR)/Artificial Neural Network (ANN), summarized a broad parametric study on composite/hybrid girders into design-ready equations, providing a clear template for converting large numerical datasets into a single practical capacity expression for multi-parameter girder design. For plates with cut-outs, Jagan Jayabalan et al.^11^ compared ANN, GEP, and EPR and again found ANN gave the best predictions. Extending elastic shear-buckling prediction for tapered webs, R. I. Shahin et al.^12^ trained an ANN on a large FE set to estimate critical coefficients, and R. I. Shahin et al.^13^ proposed a corrected coefficient from FE parametrics. In perforated tapered sections under shear, De’nan et al.^14^ showed that carefully designed elliptical openings can improve shear-buckling performance without significantly increasing the weight. For bending, De’nan et al.^15^ showed how web openings reduce flexural strength by concentrating stresses around the openings. Rodrigues et al.^16^ found that circular web openings provided better stress redistribution and higher capacity than square openings, especially at larger sizes.

Despite this progress, current design documents still address tapered members with web openings only partially. The AISC Steel Construction Manual^17^, AISC 360^18^, AISC Design Guide 02^19^, AISC Design Guide 25^20^, Eurocode 3^21^, and the Egyptian Code of Practice for Steel Construction and Bridges (ECP)^22^ remain centred on prismatic members and standard details. Applied to tapered members with openings, they can be conservative and offer limited guidance on shear capacity, bending strength, and local-buckling checks where stress redistribution and post-buckling behaviour matter.

To address this gap, a research programme on tapered plate girders with web openings has been considered for investigation by researchers from Ain Shams University. The programme included two phases: an experimental phase and a numerical phase. Although the overall research programme was conducted at Ain Shams University, the experimental test reported by Taher et al.^23^ was performed at the Structural Engineering Laboratory of El Shorouk Academy. It established the baseline behaviour of the girders using measured imperfections and considering load–deflection response, strain data, and observed failure modes. The present study represents the numerical phase of this research programme. It builds on the experimental phase as the finite element models were developed and verified against the experimental results obtained from Taher et al.^23^. After verification, a full-factorial set of 115 analyses was carried out to study the effects of panel aspect ratio, opening-to-depth ratio, tapering angle, web slenderness, and opening shape. Regarding the flange dimensions and transverse-stiffener thickness, they were constant to isolate the web-panel response. The response was evaluated using practical measures, including ultimate capacity from the load–deflection curve, stiffness retention, ductility, total strain energy, and failure location. Based on these results, an empirically calibrated shear-capacity equation was developed within the studied ranges and presented in a simple design-ready form.

## Finite element model developing and verification

The numerical work is based on the experimental programme on web-tapered plate girders with circular openings reported by Taher et al.^23^. This experimental study forms the first phase of the research programme conducted at Ain Shams University. It investigated tapered panels with different opening sizes and geometries under three-point bending. The study reported the global response, local measurements, buckling initiation, ultimate shear capacity, and main deformation and strain patterns. These experimental records are used in the present study as the benchmark for FE model verification. Full details of the tested specimens, instrumentation, and loading setup are available in Taher et al.^23^.

**Fig. 1:**
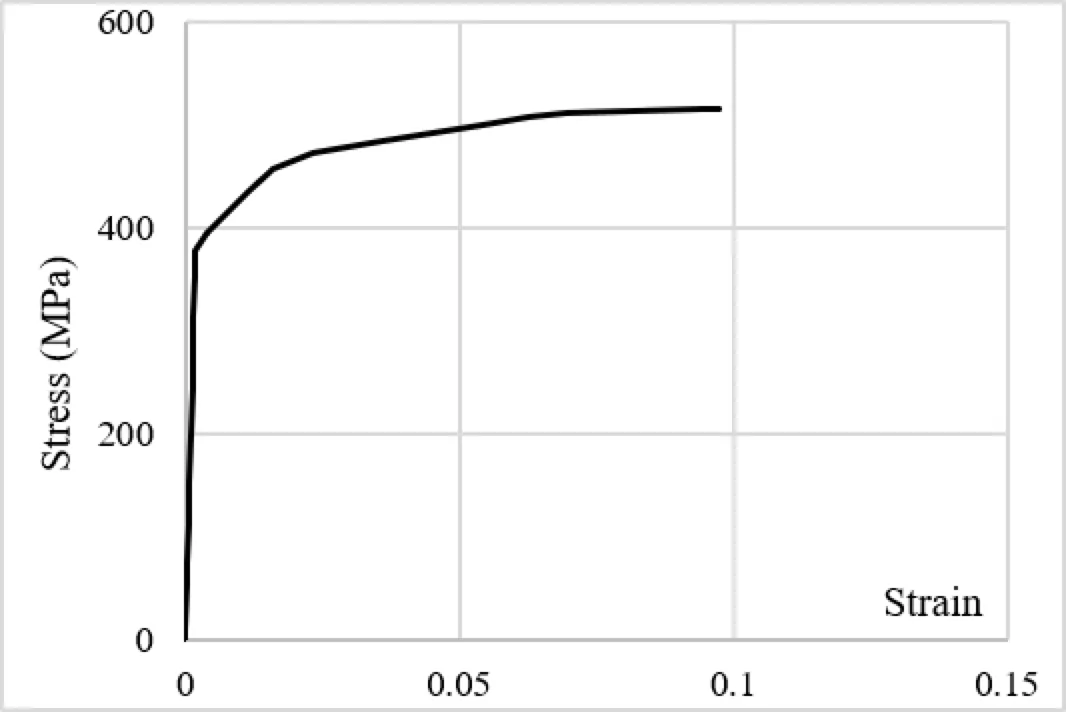
Stress–strain relationship adopted in the FE material model.

The finite element models were developed using ANSYS^24^. The webs, flanges, and transverse stiffeners were modelled using SHELL181, a four-node structural shell element from the ANSYS element library. This element type was selected to capture local shear buckling and post-buckling behaviour with reasonable computational effort. The steel material was modelled as isotropic elastic–plastic. The elastic response was defined using Young’s modulus E = 210,000 MPa, and Poisson’s ratio ν = 0.30. Yielding was based on the measured coupon-test results reported in the companion experimental study by Taher et al.^23^, with an average yield stress of approximately f_γ_ = 380 MPa and an average ultimate strength of approximately f_u_ = 530 MPa. Plastic hardening was represented using a multilinear stress–strain curve derived directly from the coupon-test stress–strain data reported in Taher et al.^23^. Only the non-descending portion of the measured curve was adopted in the ANSYS multilinear isotropic hardening model, as shown in Fig. 1.

**Fig. 2:**
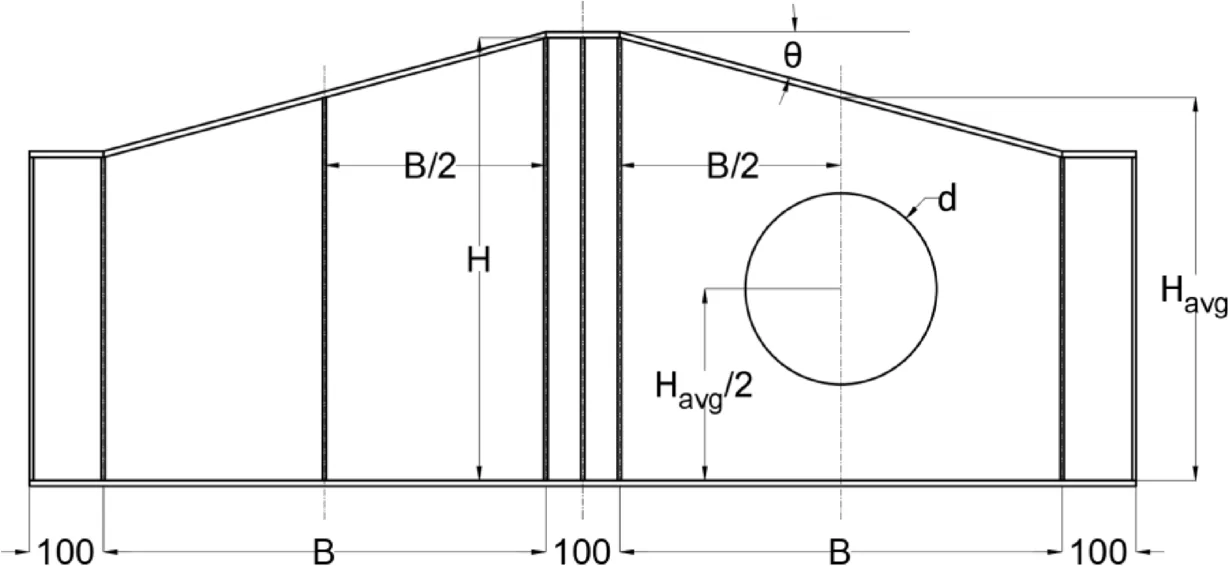
Schematic for specimen dimensions.

**Fig. 3:**
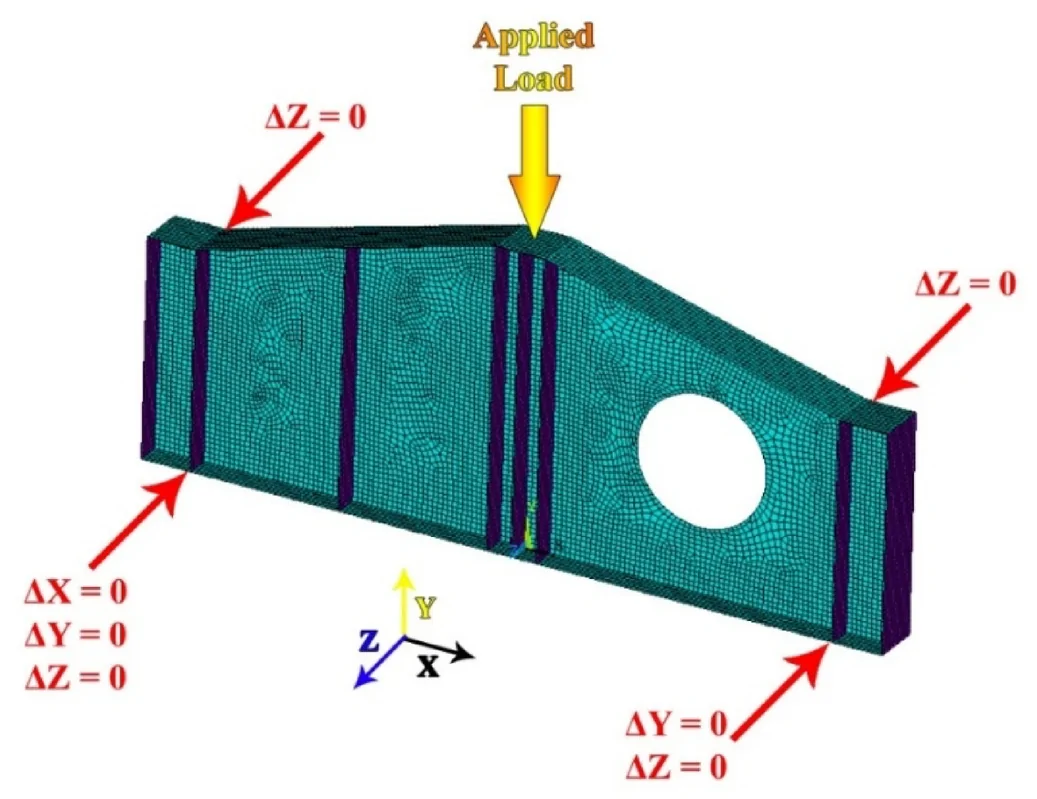
Typical model for the experimental specimens. Generated using Ansys Student 2026 R1.

**Fig. 4:**
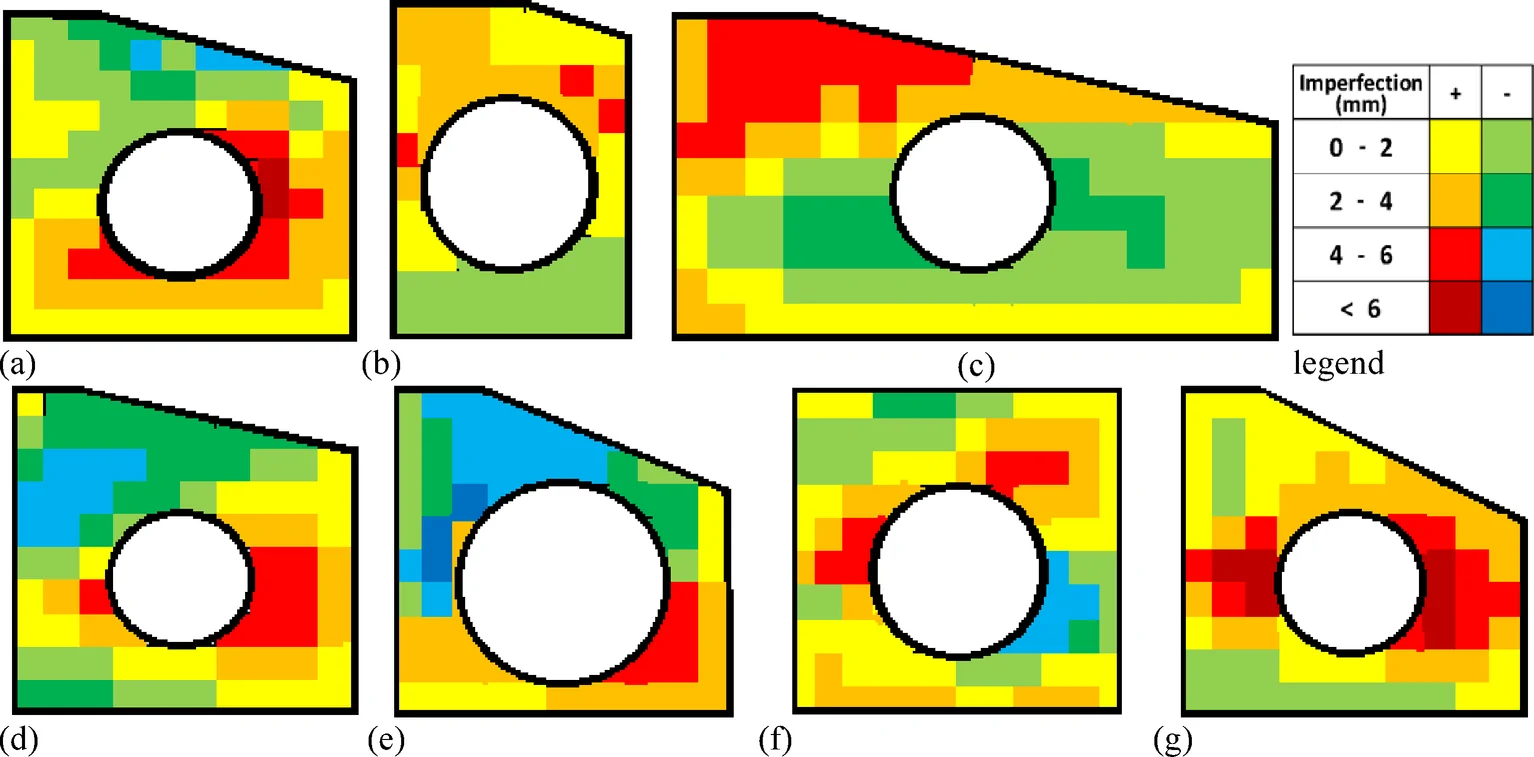
Measured out-of-flatness web panel deviation for each specimen^23^. (**a**) S1. (**b**) S2. (**c**) S3. (**d**) S4. (**e**) S5. (**f**) S6. (**g**) S7.

The model geometry followed the experimental configurations reported by Taher et al.^23^, including the measured geometric imperfections. Table 1; Fig. 2 show the geometry of the specimens. Regarding the flanges and stiffeners, their dimensions were constant in all specimens. Flanges dimensions were (100 × 8 mm) and the transverse stiffeners were 5 mm thick. A structured mesh was used, with an approximate element size of 10 mm. A mesh-sensitivity study was performed, and the selected 10 mm mesh was adopted because further mesh refinement caused only a minor change in the predicted ultimate shear capacity. The mesh-sensitivity results are summarized in Table 2 for specimen (S1). Figure 3 shows a typical finite-element model corresponding to the experimental specimens. Geometric imperfections tolerances were taken into account. The specimen-specific initial web out-of-flatness reported in Taher et al.^23^ was implemented to each specimen using nodal coordinates modifications to match the deviations of web flatness for each specimen in the finite-element models as initial imperfection fields prior to analysis, so that verification reflects the as-built geometry of each specimen. Figure 4 shows the out-of-flatness deviations of web panels measured for each specimen.

**Table 1:** Parameter values and detailed dimensions of specimens.

Specimen ID	B/H	d/H_avg_	θ	B (mm)	H (mm)	H_avg_ (mm)	d (mm)	H_avg_/t_w_
S1	1/1	1/2	15°	600	600	520	260	260
S2	2/3	1/2	15°	400	600	550	280	275
S3	3/2	1/2	15°	900	600	480	240	240
S4	1/1	1/3	15°	600	600	520	180	260
S5	1/1	2/3	15°	600	600	520	350	260
S6	1/1	1/2	0°	600	600	600	300	300
S7	1/1	1/2	25°	600	600	460	230	230

**Table 2:** Mesh sensitivity results for specimen (S1).

Test	Ultimate load (kN)
Experimental	165.08
FEM (200 mm mesh)	185.10
FEM (100 mm mesh)	176.05
FEM (10 mm mesh)	162.49
FEM (5 mm mesh)	162.68

Boundary conditions were assigned to reproduce the simply supported three-point bending test arrangement. The longitudinal, vertical, and out-of-plane directions were defined as X, Y, and Z, respectively. At one support, the vertical, lateral and longitudinal translations were restrained to prevent rigid-body motion (hinged support). At the other support, the vertical and lateral translations were restrained while the longitudinal translation was released to allow axial movement (roller support). The rotational degrees of freedom at the supports were not restrained, consistent with a simply supported condition. Additional out-of-plane restraints were applied at the bracing locations (top ends of girder) to prevent global lateral instability and ensure that web shear response governed the behaviour. The mid-span load was applied as a pressure load over the same loading area used in the experimental test setup, so that the numerical loading condition reproduced the actual load-transfer region. The applied pressure was increased incrementally during the nonlinear analysis. Static geometrically nonlinear analysis was performed with large-deformation effects activated. The arc-length method was employed to trace the nonlinear equilibrium path through local web buckling, the ultimate load, and the subsequent post-buckling response. The analysis was continued until numerical convergence could no longer be achieved.

Model outputs are mapped to the experimental observables reported by Taher et al.^23^, including load–deflection response, onset of local web buckling, ultimate shear capacity, and representative strain evolution at characteristic locations.

**Fig. 5:**
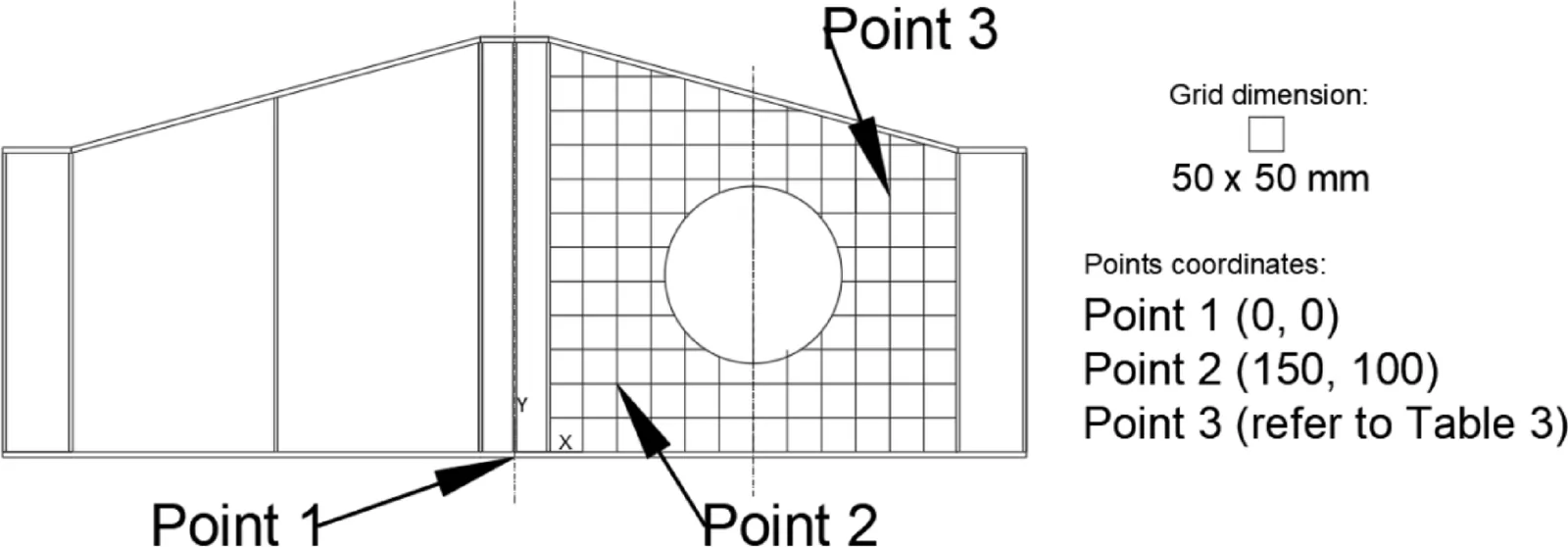
Designated locations for considered points.

**Fig. 6:**
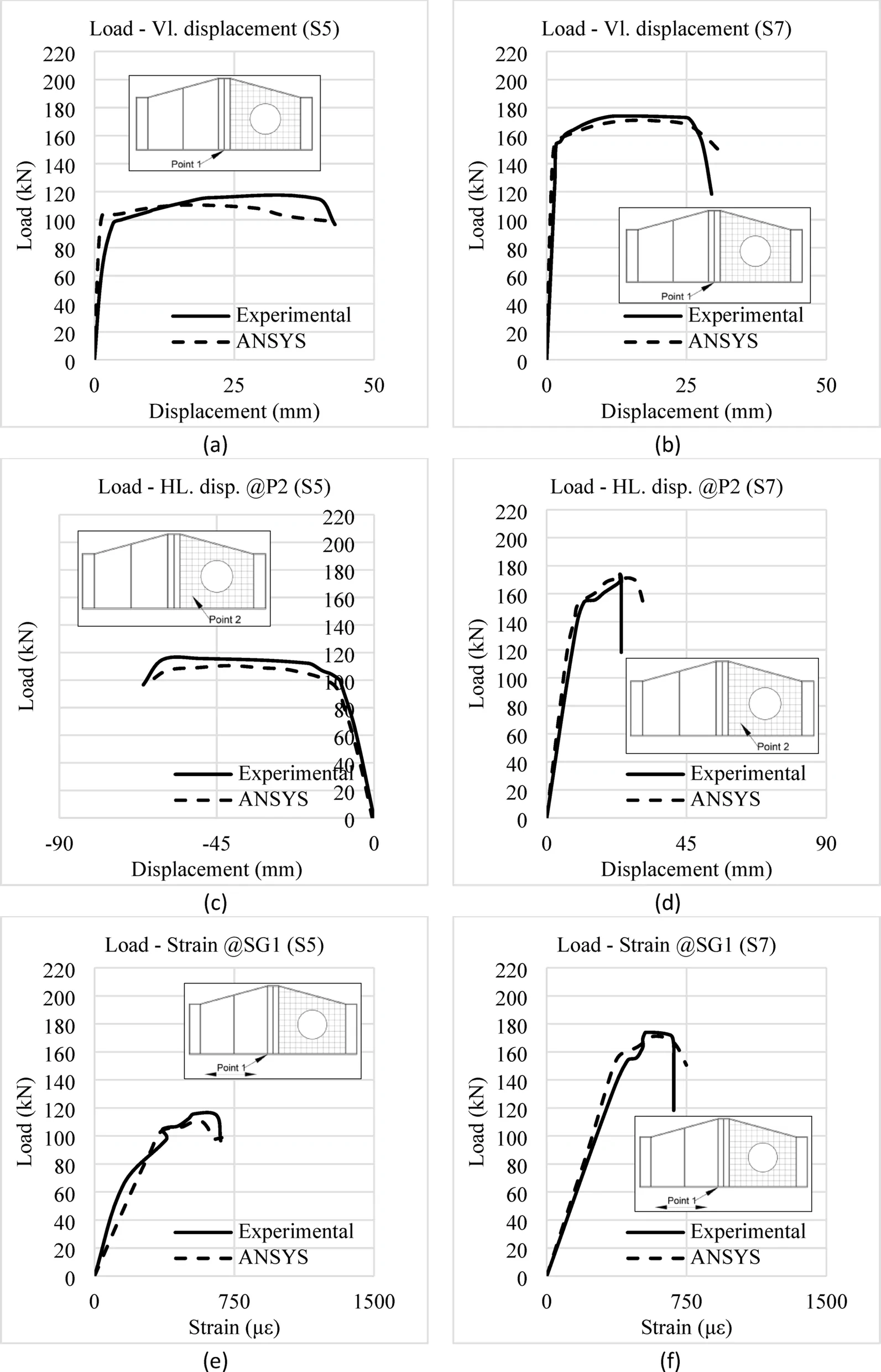

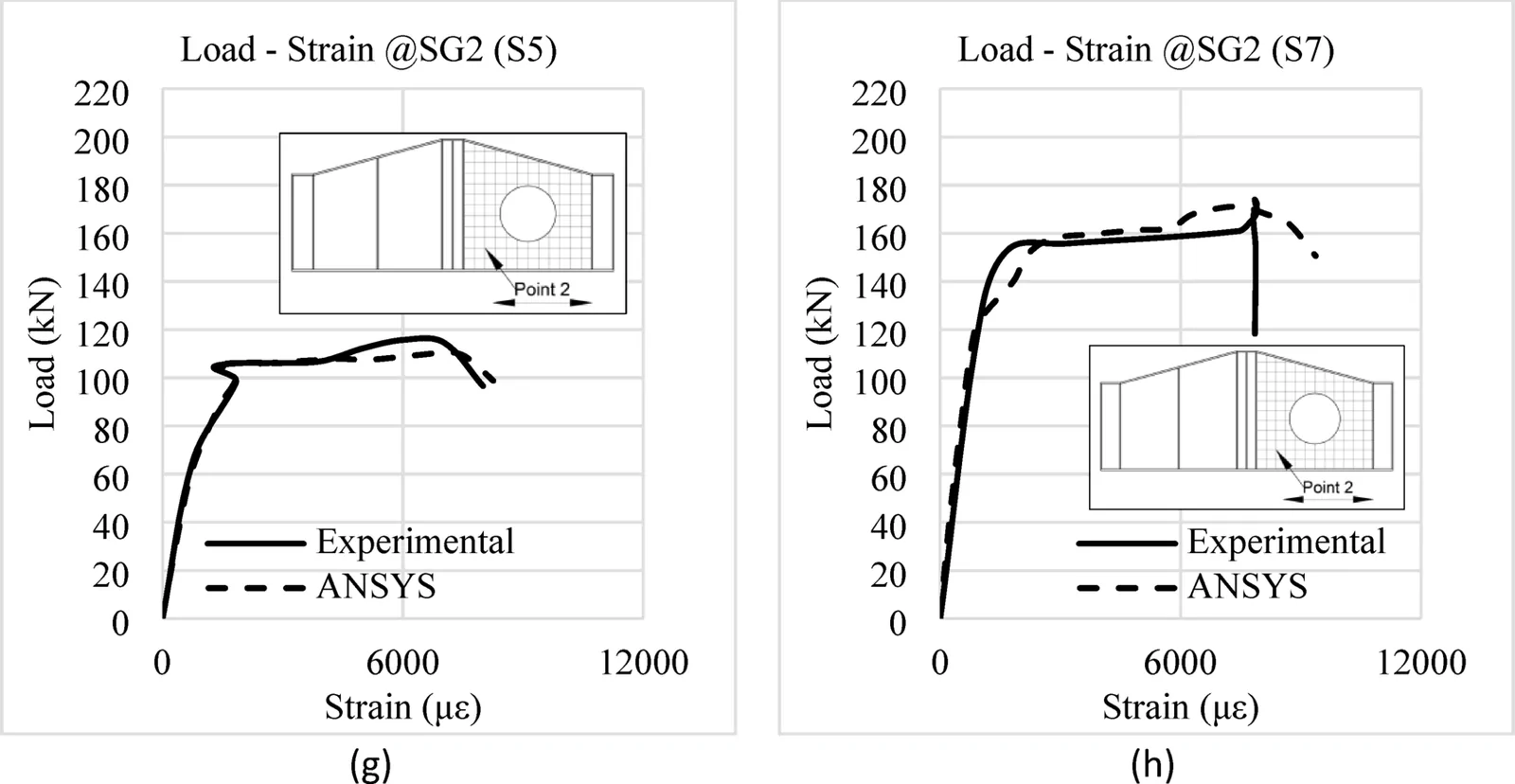
FE–experiment comparison of global and local responses for representative specimens. (**a**) Load − Mid-span deflection at point 1 for S5. (**b**) Load − Mid-span deflection at point 1 for S7. (**c**) Load−Lateral displacement at point 2 for S5. (**d**) Load−Lateral displacement at point 2 for S7. (**e**) Load−Strain at web side at point 1 for S5. (**f**) Load−Strain at web side at point 1 for S7. (**g**) Load−Strain at web side at point 2 for S5. (**h**) Load−Strain at web side at point 2 for S7.

**Fig. 7:**
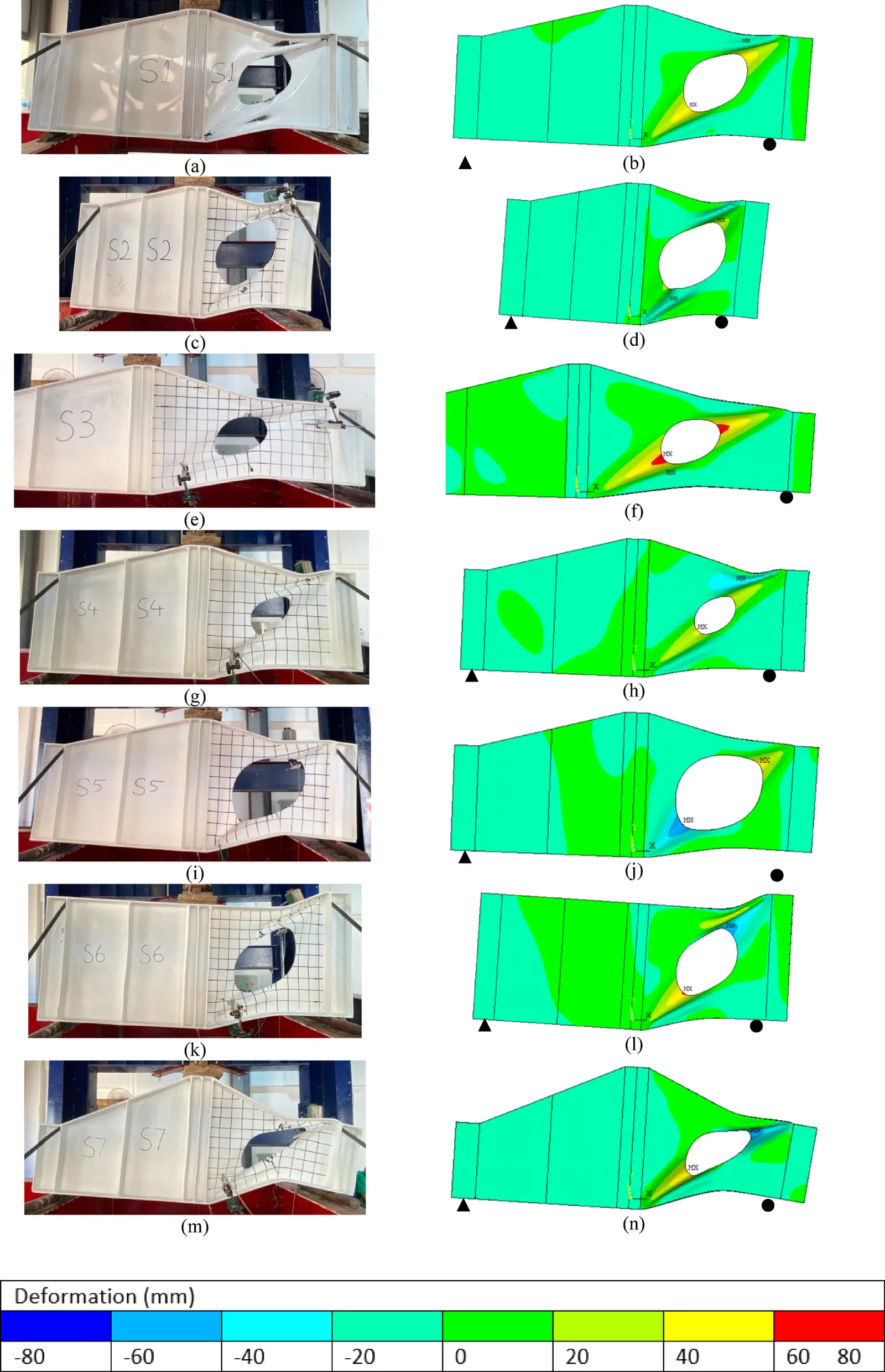
Deformed shape of specimens after test. (**a**) S1 experimental test. (**b**) S1 FEM test. (**c**) S2 experimental test. (**d**) S2 FEM test. (**e**) S3 experimental test. (**f**) S3 FEM test. (**g**) S4 experimental test. (**h**) S4 FEM test. (**i**) S5 experimental test. (**j**) S5 FEM test. (**k**) S6 experimental test. (**l**) S6 FEM test. (**m**) S7 experimental test. (**n**) S7 FEM test.

Experimental measurements at the locations illustrated in Fig. 5 and listed in Table 3 based on the work of Taher et al.^23^ were used to validate the corresponding values of the finite element model. Points 1 and 2 had fixed local coordinates for all specimens, whereas the coordinates of Point 3 varied according to the geometry of each specimen and are therefore provided separately in Table 3. Five FE–experimental response plots are used for verification at the same designated locations for instrumentations: (i) load versus mid-span vertical deflection at point 1, (ii) load versus web lateral deformation at point 2, (iii) load versus web lateral deformation at point 3, (iv) load versus flange mid-span strain at point 1, and (v) load versus web strain at point 2. Strain gauges were installed in the direction of girder axis to measure strain in that direction. The verification results show good correlation between the experimental measurements and the FEM predictions. Table 4 shows peak load and the deflection at peak load for experimental and numerical simulations with error percentages for each. In addition, representative specimens with extreme parameters values were selected to illustrate the consistency of this agreement. These comparisons are plotted in Fig. 6. Failure patterns are compared for all specimens in Fig. 7, showing agreement between the experimental observations and the FEM predictions.

**Table 3:** Point 3 coordinates for each specimen.

Specimen ID	X coordinates (mm)	Y coordinates (mm)
S1	550	375
S2	350	450
S3	800	300
S4	550	375
S5	550	375
S6	550	500
S7	500	350

**Table 4:** Experimental and numerical simulation results summary.

Specimen ID	*P*_u_experimental_	*P*_u_FEM_	ẟ_u_exp_.	ẟ_u_FEM_	Ultimate capacity error %	Ultimate deflection error %
S1	165.08	162.49	21.00	18.89	1.57	8.52
S2	195.75	202.35	17.12	15.92	3.37	7.02
S3	141.81	138.67	15.57	16.24	2.21	4.30
S4	204.45	218.80	10.75	11.79	7.02	9.67
S5	115.71	110.50	20.64	18.91	4.50	8.39
S6	159.21	155.63	17.03	18.31	2.25	7.54
S7	174.00	171.08	12.36	13.68	1.68	6.63

## Parametric study investigation

A full matrix of 115 finite-element models was used to quantify the effects of opening size, panel aspect, tapering angle, and web slenderness on the shear response. The matrix includes 81 models with circular web openings, 27 models with no web openings, and seven models with square web openings to assess opening-shape effects. The matrix investigates four dimensionless parameters over practical levels: panel aspect B/H={2/3, 1, 3/2}; opening ratio d/H_avg_={0,1/3,1/2,2/3}; tapering angle θ={0°, 15°, 25°} and web slenderness ratio is considered within the interval H_avg_/t_w_=[78, 300]. For circular openings, d denotes the opening diameter, whereas for square openings, it denotes the opening side length. The flange dimensions (100 × 8 mm) and transverse-stiffener thickness (5 mm) were kept constant throughout the study to isolate the web-panel response and all remaining modelling choices follow the verified framework in Sect. 2 and the experimental baseline reported by Taher et al.^23^. Regarding the initial geometric imperfections, they were included in all specimens. The initial imperfection shape was obtained from an elastic eigenvalue buckling analysis. The eigenmode was scaled to a maximum amplitude of approximately 8 mm, corresponding to the largest web out-of-flatness measured among the experimental specimens, which occurred in specimen S7 as shown in Fig. 4. This value was adopted as a conservative common worst-case imperfection for all parametric models. For comparison, the equivalent local geometric imperfection recommended in EN 1993-1-5 Annex C^25^ is $\:\mathrm{m}\mathrm{i}\mathrm{n}(\mathrm{a}/200,\mathrm{b}/200)$, corresponding to approximately 3 mm for a governing panel dimension of 600 mm. Therefore, the adopted measured amplitude exceeds the Eurocode reference value and provides a conservative estimation for the parametric study.

For each parameter set, the analysis tracks the load behaviour, identifies when local web buckling begins, and determines the ultimate shear capacity and corresponding mid-span deflection. The out-of-plane deformation of the web was also monitored. For the consistency of the study, all models used the same mesh density, imperfection shape and amplitude, boundary conditions, and solution procedure as those that were used in the verification.

A systematic naming convention was used for the specimens in the parametric study. Each specimen was identified by its serial number and opening shape, aspect ratio (A = B/H), diameter-to-average height ratio (R = d/H_avg_), tapering angle (θ), and web thickness (t_w_).

Specimens were numbered by opening group: circular openings first, followed by solid-web specimens, and then square openings.

The naming format is as follows:

Specimen: *Sox-Ay-Rz-u-t*.

Where:

*o* represents the opening shape, where o = C for circular opening, o = S for square opening, and o = N for no opening.

*x* represents the specimen ID/serial number, with x indicating the specimen number (e.g., 40). Accordingly, the specimen label starts with Sox (e.g., SC1, SN81, or SS109).

*Ay* represents the aspect ratio (B/H), with y indicating the ratio value (e.g., A1.0 for A = 1).

*Rz* represents the diameter-to-average height ratio (d/H_avg_), with z indicating the ratio value (e.g., R0.5 for *R* = 0.5).

*u* represents the tapering angle (θ) in degrees (e.g., 15 for θ = 15°).

*t* represents the web thickness (t) in millimeters (e.g., 2 for t = 2 mm).

**Fig. 8:**
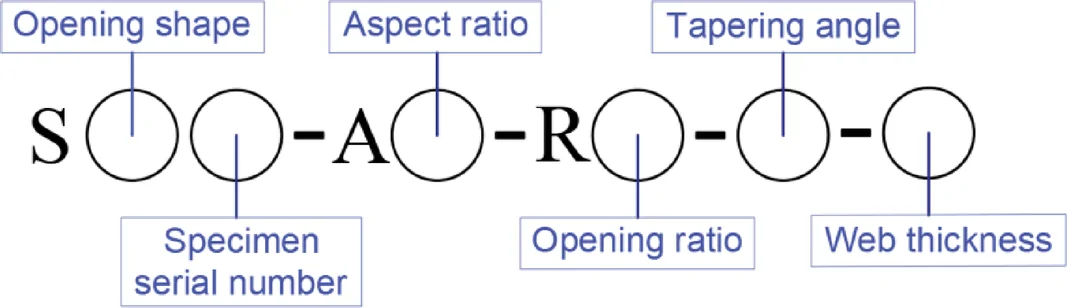
Identification of specimens names.

For example, a specimen with a circular web opening (o = C), ID number 40, aspect ratio (B/H = 1), diameter-to-average height ratio (d/H_avg_ = 0.5), tapering angle (θ = 15°) and web thickness (t_w_ = 2 mm) would be named (SC40-A1.0-R0.5–15-2). This convention provides an intuitive and concise way to identify each specimen’s geometric parameters, making it easier to reference and compare specimens throughout the study. Figure 8 illustrates the specimen identification system. Table 5 lists all 115 specimen configurations.

**Table 5:** Specimens configurations.

No.	Description (ID)	B/H	d/H_avg_	θ	Opening	t_w_	No.	Description (ID)	B/H	d/H_avg_	θ	Opening	t_w_
					Shape	(mm)						Shape	(mm)
1	SC1-A0.67-R0.33-0-2	0.67	0.33	0	Circular	2	59	SC59-A1.5-R0.33-15-3	1.5	0.33	15	Circular	3
2	SC2-A0.67-R0.33-0-3	0.67	0.33	0	Circular	3	60	SC60-A1.5-R0.33-15-5	1.5	0.33	15	Circular	5
3	SC3-A0.67-R0.33-0-5	0.67	0.33	0	Circular	5	61	SC61-A1.5-R0.33-25-2	1.5	0.33	25	Circular	2
4	SC4-A0.67-R0.33-15-2	0.67	0.33	15	Circular	2	62	SC62-A1.5-R0.33-25-3	1.5	0.33	25	Circular	3
5	SC5-A0.67-R0.33-15-3	0.67	0.33	15	Circular	3	63	SC63-A1.5-R0.33-25-5	1.5	0.33	25	Circular	5
6	SC6-A0.67-R0.33-15-5	0.67	0.33	15	Circular	5	64	SC64-A1.5-R0.5-0-2	1.5	0.5	0	Circular	2
7	SC7-A0.67-R0.33-25-2	0.67	0.33	25	Circular	2	65	SC65-A1.5-R0.5-0-3	1.5	0.5	0	Circular	3
8	SC8-A0.67-R0.33-25-3	0.67	0.33	25	Circular	3	66	SC66-A1.5-R0.5-0-5	1.5	0.5	0	Circular	5
9	SC9-A0.67-R0.33-25-5	0.67	0.33	25	Circular	5	67	SC67-A1.5-R0.5–15-2*	1.5	0.5	15	Circular	2
10	SC10-A0.67-R0.5-0-2	0.67	0.5	0	Circular	2	68	SC68-A1.5-R0.5–15-3	1.5	0.5	15	Circular	3
11	SC11-A0.67-R0.5-0-3	0.67	0.5	0	Circular	3	69	SC69-A1.5-R0.5–15-5	1.5	0.5	15	Circular	5
12	SC12-A0.67-R0.5-0-5	0.67	0.5	0	Circular	5	70	SC70-A1.5-R0.5–25-2	1.5	0.5	25	Circular	2
13	SC13-A0.67-R0.5–15-2*	0.67	0.5	15	Circular	2	71	SC71-A1.5-R0.5–25-3	1.5	0.5	25	Circular	3
14	SC14-A0.67-R0.5–15-3	0.67	0.5	15	Circular	3	72	SC72-A1.5-R0.5–25-5	1.5	0.5	25	Circular	5
15	SC15-A0.67-R0.5–15-5	0.67	0.5	15	Circular	5	73	SC73-A1.5-R0.67-0-2	1.5	0.67	0	Circular	2
16	SC16-A0.67-R0.5–25-2	0.67	0.5	25	Circular	2	74	SC74-A1.5-R0.67-0-3	1.5	0.67	0	Circular	3
17	SC17-A0.67-R0.5–25-3	0.67	0.5	25	Circular	3	75	SC75-A1.5-R0.67-0-5	1.5	0.67	0	Circular	5
18	SC18-A0.67-R0.5–25-5	0.67	0.5	25	Circular	5	76	SC76-A1.5-R0.67-15-2	1.5	0.67	15	Circular	2
19	SC19-A0.67-R0.67-0-2	0.67	0.67	0	Circular	2	77	SC77-A1.5-R0.67-15-3	1.5	0.67	15	Circular	3
20	SC20-A0.67-R0.67-0-3	0.67	0.67	0	Circular	3	78	SC78-A1.5-R0.67-15-5	1.5	0.67	15	Circular	5
21	SC21-A0.67-R0.67-0-5	0.67	0.67	0	Circular	5	79	SC79-A1.5-R0.67-25-2	1.5	0.67	25	Circular	2
22	SC22-A0.67-R0.67-15-2	0.67	0.67	15	Circular	2	80	SC80-A1.5-R0.67-25-3	1.5	0.67	25	Circular	3
23	SC23-A0.67-R0.67-15-3	0.67	0.67	15	Circular	3	81	SC81-A1.5-R0.67-25-5	1.5	0.67	25	Circular	5
24	SC24-A0.67-R0.67-15-5	0.67	0.67	15	Circular	5	82	SN82-A0.67-R0-0–2	0.67	0	0	N/A	2
25	SC25-A0.67-R0.67-25-2	0.67	0.67	25	Circular	2	83	SN83-A0.67-R0-0–3	0.67	0	0	N/A	3
26	SC26-A0.67-R0.67-25-3	0.67	0.67	25	Circular	3	84	SN84-A0.67-R0-0–5	0.67	0	0	N/A	5
27	SC27-A0.67-R0.67-25-5	0.67	0.67	25	Circular	5	85	SN85-A0.67-R0-15-2	0.67	0	15	N/A	2
28	SC28-A1-R0.33-0-2	1	0.33	0	Circular	2	86	SN86-A0.67-R0-15-3	0.67	0	15	N/A	3
29	SC29-A1-R0.33-0-3	1	0.33	0	Circular	3	87	SN87-A0.67-R0-15-5	0.67	0	15	N/A	5
30	SC30-A1-R0.33-0-5	1	0.33	0	Circular	5	88	SN88-A0.67-R0-25-2	0.67	0	25	N/A	2
31	SC31-A1-R0.33-15-2*	1	0.33	15	Circular	2	89	SN89-A0.67-R0-25-3	0.67	0	25	N/A	3
32	SC32-A1-R0.33-15-3	1	0.33	15	Circular	3	90	SN90-A0.67-R0-25-5	0.67	0	25	N/A	5
33	SC33-A1-R0.33-15-5	1	0.33	15	Circular	5	91	SN91-A1-R0-0–2	1	0	0	N/A	2
34	SC34-A1-R0.33-25-2	1	0.33	25	Circular	2	92	SN92-A1-R0-0–3	1	0	0	N/A	3
35	SC35-A1-R0.33-25-3	1	0.33	25	Circular	3	93	SN93-A1-R0-0–5	1	0	0	N/A	5
36	SC36-A1-R0.33-25-5	1	0.33	25	Circular	5	94	SN94-A1-R0-15-2	1	0	15	N/A	2
37	SC37-A1-R0.5-0-2*	1	0.5	0	Circular	2	95	SN95-A1-R0-15-3	1	0	15	N/A	3
38	SC38-A1-R0.5-0-3	1	0.5	0	Circular	3	96	SN96-A1-R0-15-5	1	0	15	N/A	5
39	SC39-A1-R0.5-0-5	1	0.5	0	Circular	5	97	SN97-A1-R0-25-2	1	0	25	N/A	2
40	SC40-A1-R0.5–15-2*	1	0.5	15	Circular	2	98	SN98-A1-R0-25-3	1	0	25	N/A	3
41	SC41-A1-R0.5–15-3	1	0.5	15	Circular	3	99	SN99-A1-R0-25-5	1	0	25	N/A	5
42	SC42-A1-R0.5–15-5	1	0.5	15	Circular	5	100	SN100-A1.5-R0-0–2	1.5	0	0	N/A	2
43	SC43-A1-R0.5–25-2*	1	0.5	25	Circular	2	101	SN101-A1.5-R0-0–3	1.5	0	0	N/A	3
44	SC44-A1-R0.5–25-3	1	0.5	25	Circular	3	102	SN102-A1.5-R0-0–5	1.5	0	0	N/A	5
45	SC45-A1-R0.5–25-5	1	0.5	25	Circular	5	103	SN103-A1.5-R0-15-2	1.5	0	15	N/A	2
46	SC46-A1-R0.67-0-2	1	0.67	0	Circular	2	104	SN104-A1.5-R0-15-3	1.5	0	15	N/A	3
47	SC47-A1-R0.67-0-3	1	0.67	0	Circular	3	105	SN105-A1.5-R0-15-5	1.5	0	15	N/A	5
48	SC48-A1-R0.67-0-5	1	0.67	0	Circular	5	106	SN106-A1.5-R0-25-2	1.5	0	25	N/A	2
49	SC49-A1-R0.67-15-2*	1	0.67	15	Circular	2	107	SN107-A1.5-R0-25-3	1.5	0	25	N/A	3
50	SC50-A1-R0.67-15-3	1	0.67	15	Circular	3	108	SN108-A1.5-R0-25-5	1.5	0	25	N/A	5
51	SC51-A1-R0.67-15-5	1	0.67	15	Circular	5	109	SS109-A0.67-R0.5–15-2	0.67	0.5	15	Squared	2
52	SC52-A1-R0.67-25-2	1	0.67	25	Circular	2	110	SS110-A1-R0.33-15-2	1	0.33	15	Squared	2
53	SC53-A1-R0.67-25-3	1	0.67	25	Circular	3	111	SS111-A1-R0.5-0-2	1	0.5	0	Squared	2
54	SC54-A1-R0.67-25-5	1	0.67	25	Circular	5	112	SS112-A1-R0.5–15-2	1	0.5	15	Squared	2
55	SC55-A1.5-R0.33-0-2	1.5	0.33	0	Circular	2	113	SS113-A1-R0.5–25-2	1	0.5	25	Squared	2
56	SC56-A1.5-R0.33-0-3	1.5	0.33	0	Circular	3	114	SS114-A1-R0.67-15-2	1	0.67	15	Squared	2
57	SC57-A1.5-R0.33-0-5	1.5	0.33	0	Circular	5	115	SS115-A1.5-R0.5–15-2	1.5	0.5	15	Squared	2
58	SC58-A1.5-R0.33-15-2	1.5	0.33	15	Circular	2	*FE models verified against corresponding experimental specimens						

## Response characterization

###  Ultimate shear capacity (V_u_)

**Fig. 9:**
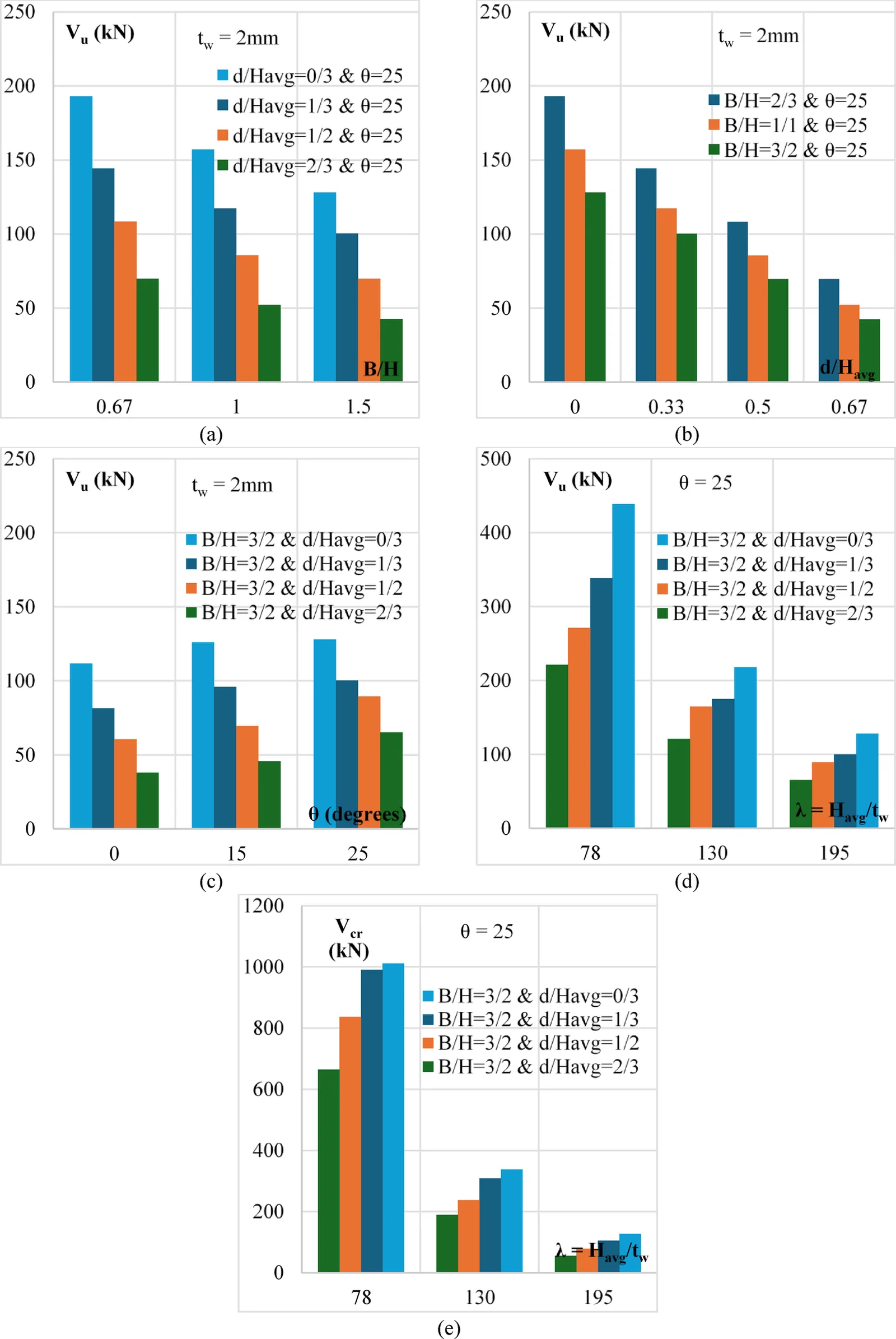
Influence of parameters on shear capacity. (**a**) influence of aspect ratio on V_u_. (**b**) influence of opening diameter ratio on V_u_. (**c**) influence of tapering angle on V_u_. (**d**) influence of slenderness ratio on V_u_. (**e**) influence of slenderness ratio on V_cr_.

To illustrate the effect of the main parameters, representative specimens from the parametric study are presented in Fig. 9 using the most critical value regarding each parameter. The ultimate shear capacity $\:{\hspace{0.17em}}{\mathrm{V}}_{\mathrm{u}}$ is taken as the maximum value reached in the numerical response before any sustained drop in resistance. In general, $\:{\mathrm{V}}_{\mathrm{u}}$ decreases as the panel aspect ratio $\:\mathrm{B}/\mathrm{H}$ increases, with an average reduction of about 30–40% from the lowest to the highest $\:\mathrm{B}/\mathrm{H}$ level as shown in Fig. 9a. A much stronger drop is observed with increasing opening ratio $\:\mathrm{d}/{\mathrm{H}}_{\mathrm{a}\mathrm{v}\mathrm{g}}$, where moving from no opening to $\:\mathrm{d}/{\mathrm{H}}_{\mathrm{a}\mathrm{v}\mathrm{g}}=2/3$ leads to an average reduction of about 60–70% in $\:{\mathrm{V}}_{\mathrm{u}}$, as shown in Fig. 9b. In contrast, increasing the tapering angle $\:{\uptheta\:}$ shows a beneficial effect, with $\:{\mathrm{V}}_{\mathrm{u}}$ typically increasing by about 15%, and this improvement becomes more noticeable at larger opening ratios as seen in Fig. 9c. All web thicknesses showed the same trend but $\:{\mathrm{t}}_{\mathrm{w}}=2$mm specimens were selected for trend figures because they represent the most slender and critical cases. Web slenderness has the most pronounced influence, where increasing $\:{\uplambda\:}$ ($\:{\mathrm{H}}_{\mathrm{a}\mathrm{v}\mathrm{g}}/{\mathrm{t}}_{\mathrm{w}})$ over the investigated range reduces $\:{\mathrm{V}}_{\mathrm{u}}$ by about 70–75% on average due to earlier web buckling and a reduced post-buckling reserve as can be seen in Fig. 9d. In addition, the influence of slenderness in critical shear capacity ($\:{\mathrm{V}}_{\mathrm{c}\mathrm{r}}$) is shown in Fig. 9e. It shows that increasing the web slenderness $\:{\uplambda\:}$from 78 to 195 reduces the critical shear capacity by approximately 88–92%.

### Failure modes

For clarity, three failure modes were used to classify the numerical results. Failure mode 1 (FM1) refers to web-controlled elastic–plastic failure, marked by local web buckling which led to stress concentrations around the web opening. Failure mode 2 (FM2) refers to yielding of the compression flange at mid-span. Failure mode 3 (FM3) refers to local buckling of the compression flange.

**Fig. 10:**
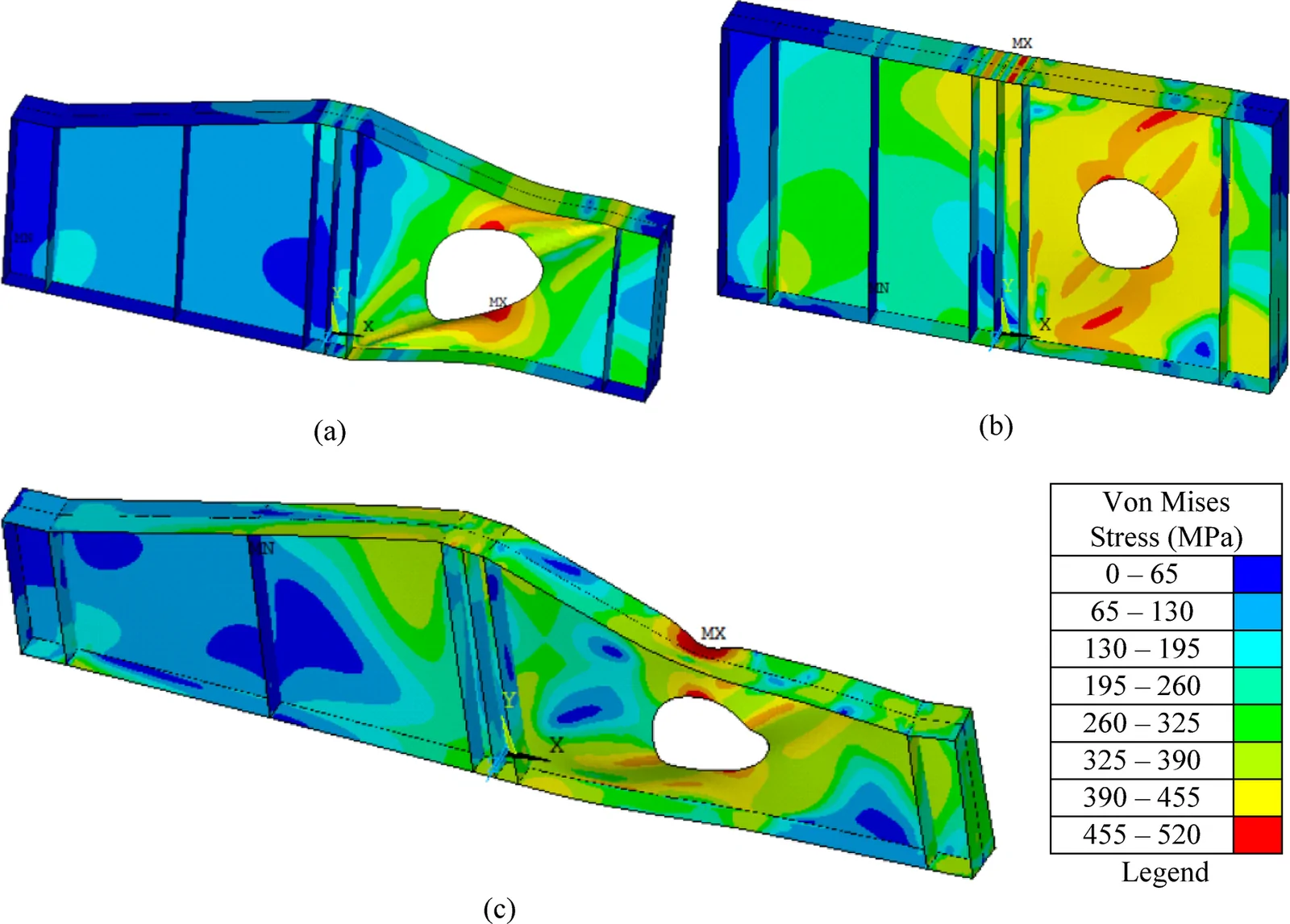
Representative different failure modes of specimens with Von Mises stress distribution. (**a**) Web-panel local buckling with elasto-plastic spread (FM1). (**b**) Compression-flange yielding at mid-span (FM2). (**c**) Compression-flange local buckling (FM3). Generated using Ansys Student 2026 R1.

**Fig. 11:**
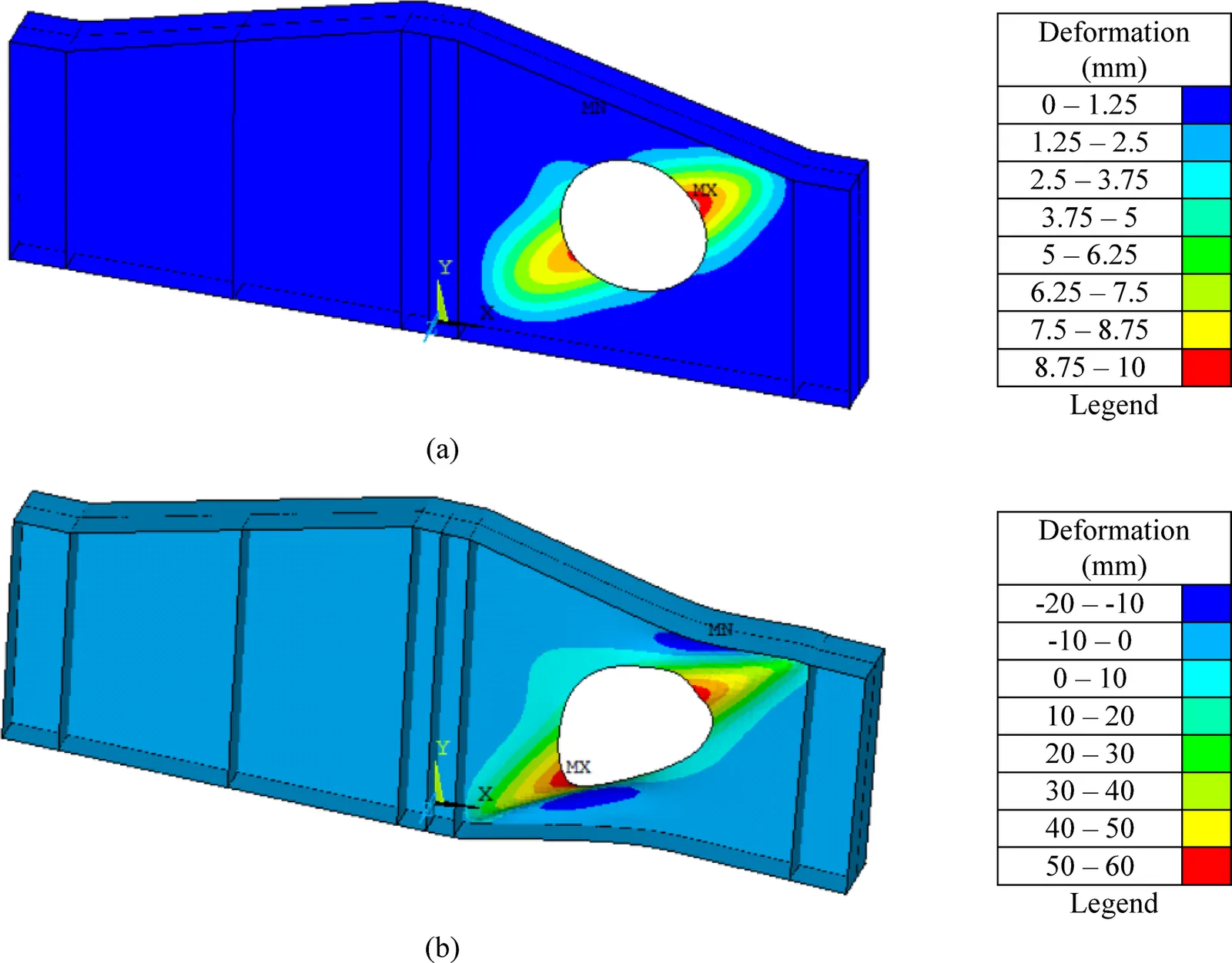
Lateral deformations of (SC40*-A1-R0.5–15-2). (**a**) At the initiation of local buckling. (**b**) At failure. Generated using Ansys Student 2026 R1.

Most specimens failed in FM1. In this mode, local out-of-plane buckling formed in the web panel, often around the opening, producing stress concentrations in limited regions around the web opening, as shown by the red zones in the specimen stress distributions in Fig. 10a. FM1 dominated for $\:{\mathrm{t}}_{\mathrm{w}}=2$mm and $\:{\mathrm{t}}_{\mathrm{w}}=3$mm over most of the investigated ranges, showing that the response was governed mainly by web instability. Figure 11 shows an FM1 deformation pattern at the initiation of local buckling and at failure.

The non-web failure modes, FM2 and FM3, appeared only in limited parameter combinations. FM2 was observed in 8 specimens. It occurred in specimens with $\:\mathrm{B}/\mathrm{H}=0.67$, $\:{\mathrm{t}}_{\mathrm{w}}=5$mm, and $\:\mathrm{d}/{\mathrm{H}}_{\mathrm{a}\mathrm{v}\mathrm{g}}=0$ or * 0.33* for all investigated tapering angles. It also occurred in the solid-web specimens with $\:\mathrm{B}/\mathrm{H}=1.0$, $\:{\mathrm{t}}_{\mathrm{w}}=5$mm, and $\:{\uptheta\:}={0}^{\circ\:}$ or $\:{15}^{\circ\:}$. These cases show that the web remained strong enough to prevent web-controlled failure, so the response shifted to yielding of the compression flange.

FM3 was observed in 10 specimens. It occurred in the solid-web specimens with $\:\mathrm{B}/\mathrm{H}=1.5$ and $\:{\mathrm{t}}_{\mathrm{w}}=5$mm for all investigated tapering angles, in the specimens with $\:\mathrm{B}/\mathrm{H}=1.5$, $\:{\mathrm{t}}_{\mathrm{w}}=5$mm, and $\:{\uptheta\:}={25}^{\circ\:}$ for all investigated opening ratios, in the solid-web specimens with $\:\mathrm{B}/\mathrm{H}=1.0$ and $\:{\mathrm{t}}_{\mathrm{w}}=3$mm for all investigated tapering angles, and in the solid-web specimen with $\:\mathrm{B}/\mathrm{H}=1.0$, $\:{\mathrm{t}}_{\mathrm{w}}=5$mm, and $\:{\uptheta\:}={25}^{\circ\:}$. These cases indicate an increased tendency for local instability of the compression flange.

The solid-web group with $\:\mathrm{B}/\mathrm{H}=1.0$ is especially useful in explaining the shift between FM2 and FM3. For $\:{\mathrm{t}}_{\mathrm{w}}=5$mm, the mode was FM2 at $\:{\uptheta\:}={0}^{\circ\:}$ and $\:{15}^{\circ\:}$, but changed to FM3 at $\:{\uptheta\:}={25}^{\circ\:}$. When the web thickness was reduced to $\:{\mathrm{t}}_{\mathrm{w}}=3$mm, the same case showed FM3 at all tapering angles. This suggests that the 3 mm web did not provide enough restraint to the compression flange, while the 5 mm web allowed flange yielding at the lower tapering angles. At $\:{\uptheta\:}={25}^{\circ\:}$, the larger taper reduced the web depth more significantly and shifted the critical stress toward the flange region above the web opening, where local buckling became more critical than yielding.

Figure 10b,c present representative FM2 and FM3 specimens respectively. The specimens that exhibited non-web failure modes, FM2 and FM3 are summarized in Table 6. These specimens are excluded from the shear capacity formulations since the failure didn’t occur in web to keep consistency.

**Table 6:** Specimens and corresponding failure mode.

No.	Description (ID)	Failure mode	No.	Description (ID)	Failure mode
1	SC3-A0.67-R0.33-0-5	FM2	10	SC72-A1.5-R0.5–25-5	FM3
2	SC6-A0.67-R0.33-15-5	FM2	11	SC81-A1.5-R0.67-25-5	FM3
3	SC9-A0.67-R0.33-25-5	FM2	12	SN92-A1-R0-0–3	FM3
4	SN84-A0.67-R0-0–5	FM2	13	SN95-A1-R0-15-3	FM3
5	SN87-A0.67-R0-15-5	FM2	14	SN98-A1-R0-25-3	FM3
6	SN90-A0.67-R0-25-5	FM2	15	SN99-A1-R0-25-5	FM3
7	SN93-A1-R0-0–5	FM2	16	SN102-A1.5-R0-0–5	FM3
8	SN96-A1-R0-15-5	FM2	17	SN105-A1.5-R0-15-5	FM3
9	SC63-A1.5-R0.33-25-5	FM3	18	SN108-A1.5-R0-25-5	FM3

*All remaining specimens exhibited FM1.

### Effect of opening shape

**Fig. 12:**
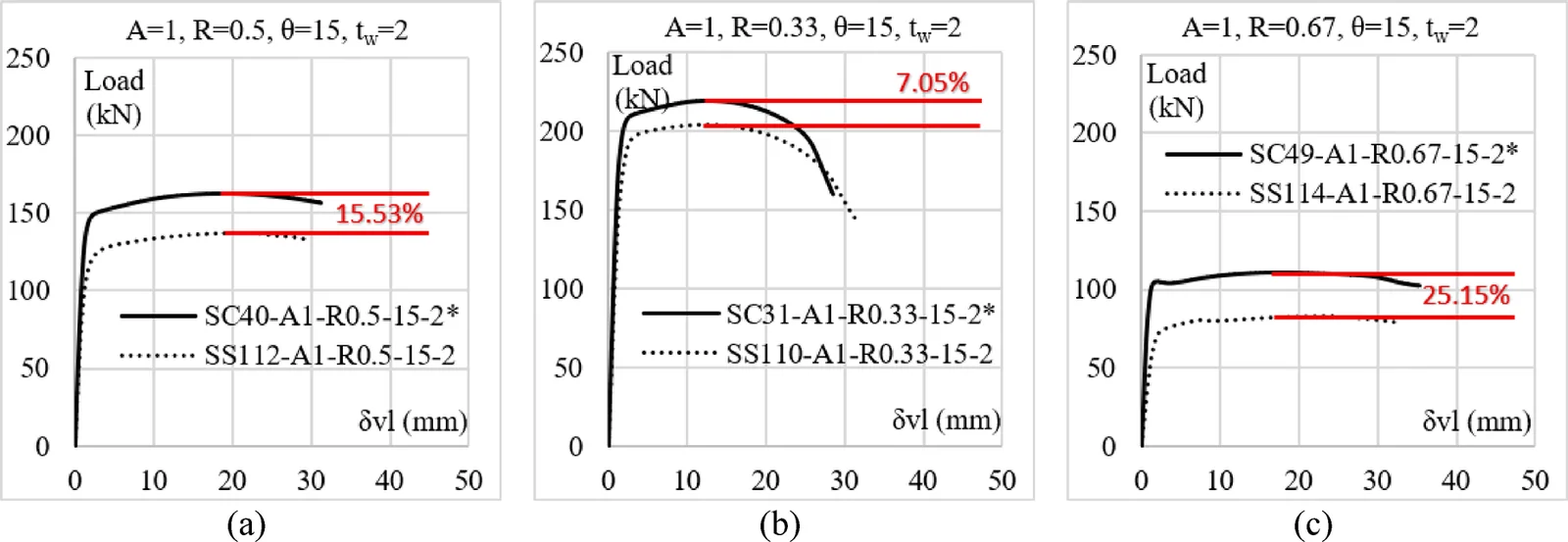
Comparison between circular and square openings (representative FE comparisons). (**a**) specimens with (A = 1, *R* = 0.5, θ = 15, t_w_=2). (**b**) specimens with (A = 1, *R* = 0.33, θ = 15, t_w_=2). (**c**) specimens with (A = 1, *R* = 0.67, θ = 15, t_w_=2).

To assess the effect of opening shape, seven additional FE models with square openings were analysed. Their parameter values were selected to match the seven experimentally tested specimens, and each square-opening model was paired with a geometrically identical model containing a circular opening (same $\:\mathrm{B}/\mathrm{H}$, $\:\mathrm{d}/{\mathrm{H}}_{\mathrm{avg}},$ $\:{\uptheta}$, and $\:{\mathrm{t}}_{\mathrm{w}}$). These specimens’ geometry details are shown in Table 1 except that they are made with square openings instead of circular where d here denotes the opening side length. All modelling assumptions follow the verified framework in Sect. 2. The FE–FE comparisons are illustrated in Fig. 12 using representative matched pairs, selected to demonstrate the consistent differences observed among specimens with different diameter ratios ($\:\mathrm{d}/{\mathrm{H}}_{\mathrm{avg}}$).

Across all matched pairs, the specimens with circular openings generally achieved higher ultimate shear capacity than their square-opening counterparts. The increase in ultimate shear capacity ranged from approximately 7% to 25% across the investigated specimens. Moreover, the effect of opening shape became more noticeable as the opening size increased. Large square openings caused a greater reduction in shear capacity than circular openings because their sharp corners concentrated stresses and deformation around the opening. This is consistent with previous studies on perforated steel plates and steel beams with web openings, where openings with sharp corners were shown to produce higher stress concentrations and more localized deformation than rounded openings, particularly when the opening is relatively large^16^.

### Initial (elastic) stiffness (K_i_)

**Fig. 13:**
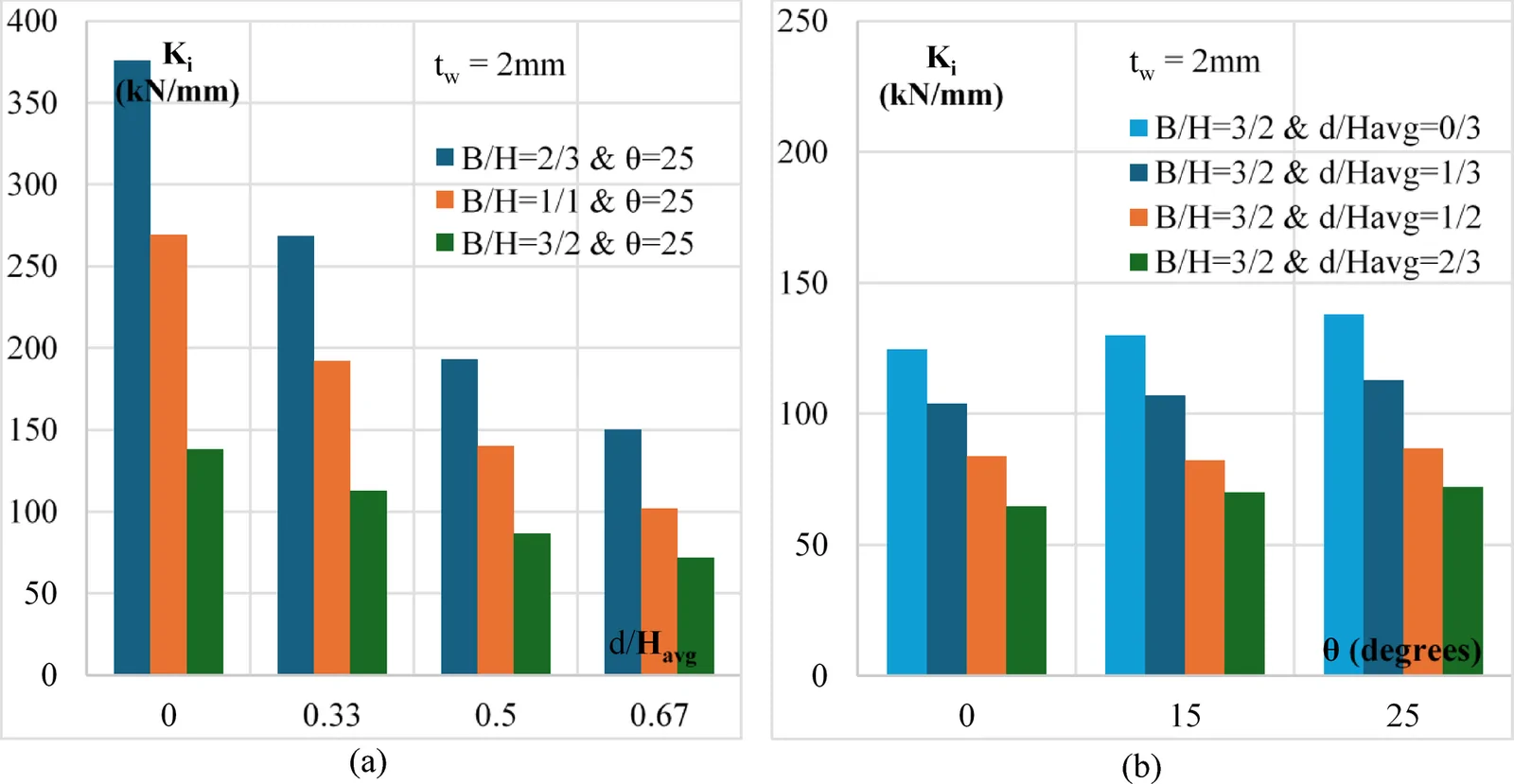
Influence of parameters on K_i_. (**a**) influence of opening diameter ratio. (**b**) influence of tapering angle.

Initial stiffness $\:{\mathrm{K}}_{\mathrm{i}}$ was calculated from the slope of the initial linear part of the load–deflection curve. This value represents the elastic resistance of the girder before web buckling starts. Representative specimens from the parametric study are presented in Fig. 13 to show the effect of the main independent parameters.

The effects of panel aspect ratio and web slenderness are not discussed separately in this section. Both parameters are already reflected in the elastic stiffness through the girder geometry and shear rigidity.

The opening ratio $\:\mathrm{d}/{\mathrm{H}}_{\mathrm{a}\mathrm{v}\mathrm{g}}$ had the clearest effect on $\:{\mathrm{K}}_{\mathrm{i}}$. As the opening ratio increased, the initial stiffness decreased in a consistent manner. Within the studied range, this reduction was about 40–55%, as shown in Fig. 13a. This is mainly due to the reduction in the effective web area, which decreases the shear rigidity during the elastic stage.

The effect of the tapering angle ($\:{\uptheta\:}$) was less pronounced. Increasing $\:{\uptheta\:}$ from 0° to 25° changed $\:{\mathrm{K}}_{\mathrm{i}}$ by about 10%, as shown in Fig. 13b. This indicates that tapering mainly affects how stiffness is distributed along the web rather than the overall elastic response. In general, the initial stiffness is controlled mainly by the opening configuration, while the tapering angle has a smaller effect within the studied range.

### Beam ductility (δ_u_/δ_e_)

**Fig. 14:**
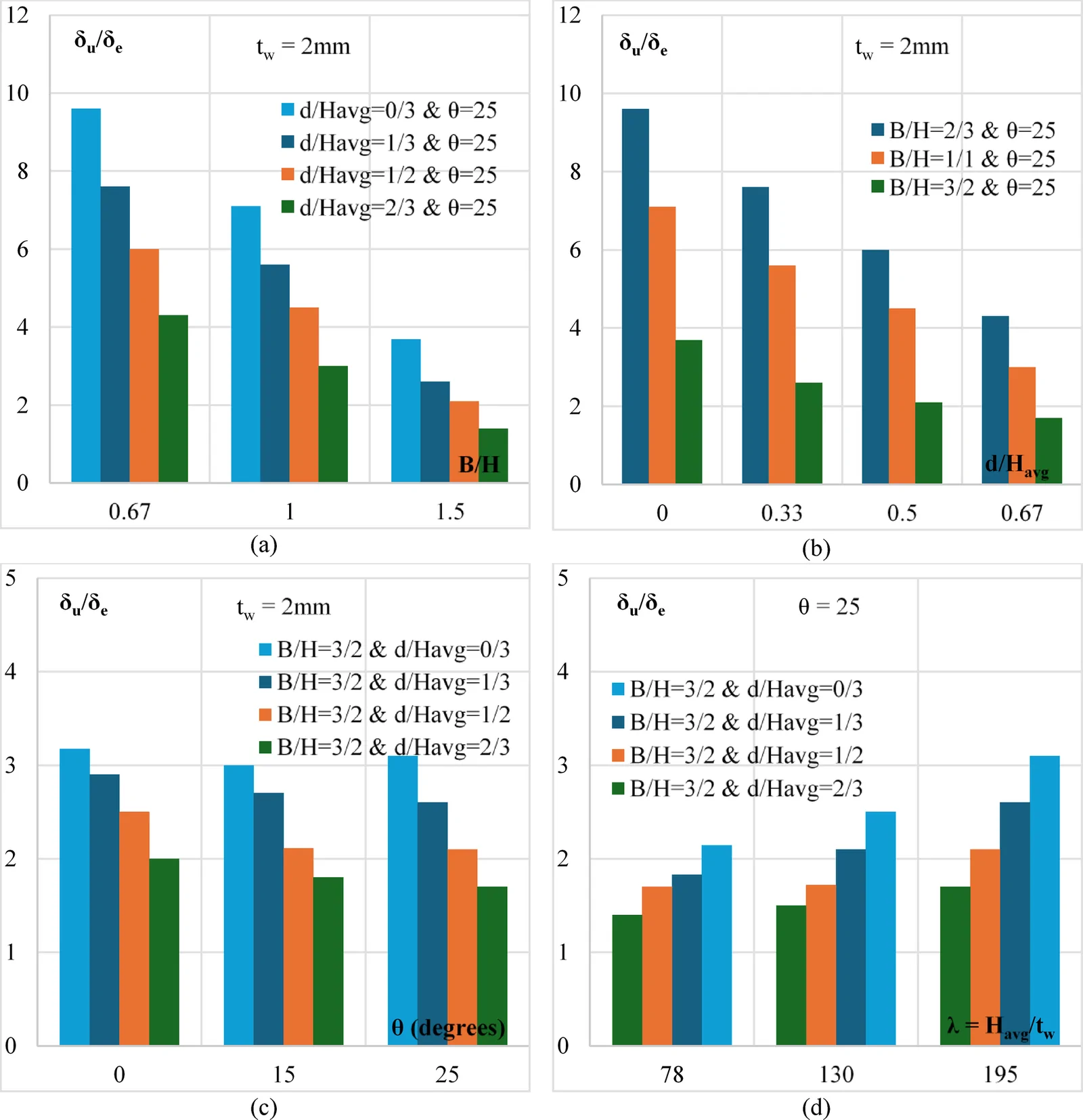
Influence of parameters on ductility. (**a**) influence of aspect ratio. (**b**) influence of opening diameter ratio. (**c**) influence of tapering angle. (**d**) influence of slenderness ratio.

Ductility was evaluated using the displacement ratio $\:{{\updelta\:}}_{\mathrm{u}}/{{\updelta\:}}_{\mathrm{e}}$, which represents the deformation capacity available beyond the elastic stage up to the ultimate state. Figure 14 illustrates the influence of the investigated parameters on this ratio using representative specimens from the parametric study. As shown in Fig. 14a, increasing the panel aspect ratio $\:\mathrm{B}/\mathrm{H}$ reduces the ductility by approximately 60–70%, indicating that longer panels fail with a smaller post-buckling deformation reserve. A similar trend is observed for the opening ratio $\:\mathrm{d}/{\mathrm{H}}_{\mathrm{a}\mathrm{v}\mathrm{g}}$, where increasing the opening size decreases the ductility by approximately 50–60% owing to the reduction in effective web stiffness (Fig. 14b). In contrast, the tapering angle has only a limited influence, producing variations of approximately 10% over the investigated range (Fig. 14c). The web slenderness ratio exhibits an opposite trend (Fig. 14d); increasing $\:{\mathrm{H}}_{\mathrm{a}\mathrm{v}\mathrm{g}}/{\mathrm{t}}_{\mathrm{w}}$ increases the ductility ratio by approximately 20–40%. This increase should not be interpreted as improved structural performance, but rather as a consequence of the earlier onset of buckling, which reduces the elastic displacement $\:{{\updelta\:}}_{\mathrm{e}}$ more rapidly than the ultimate displacement $\:{{\updelta\:}}_{\mathrm{u}}$. Overall, the panel aspect ratio, opening ratio, and web slenderness exert the greatest influence on ductility, whereas the tapering angle has only a minor effect within the investigated parameter range.

### Total strain energy of beams

**Fig. 15:**
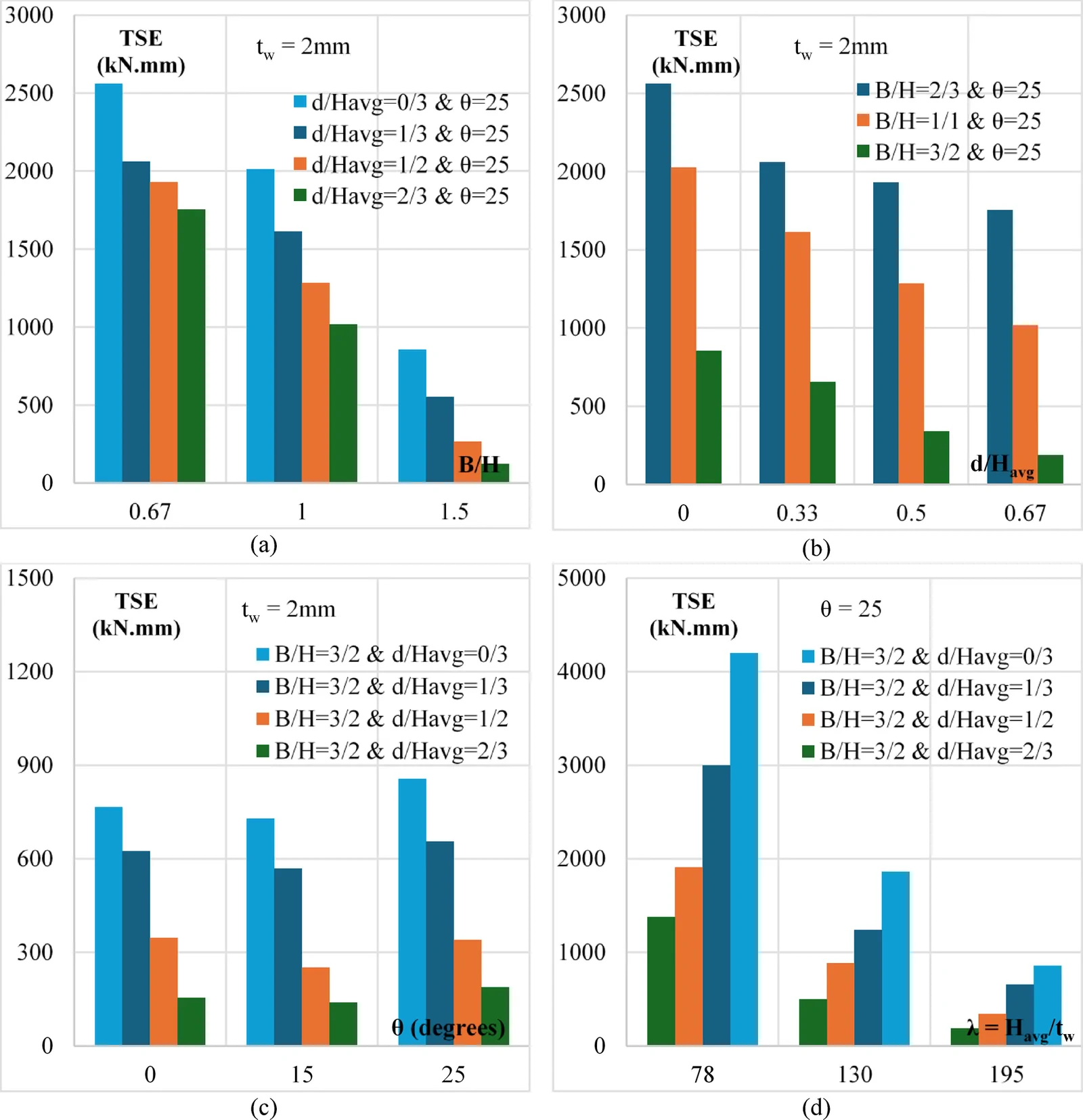
Influence of parameters on total strain energy. (**a**) influence of aspect ratio. (**b**) influence of opening diameter ratio. (**c**) influence of tapering angle. (**d**) influence of slenderness ratio.

The Total Strain Energy (TSE) was calculated as the area under the load–deflection curve up to the ultimate state, using the mid-span vertical deflection as the displacement response. To illustrate the influence of the main parameters, Fig. 15 presents representative specimens selected from the parametric matrix, in which peak values of the investigated parameters are used to capture the governing trends of the study. The total strain energy (TSE) response varies with the parameters investigated. On average, increasing the panel aspect ratio $\:\mathrm{B}/\mathrm{H}$ from the lowest to the highest value leads to a reduction in total strain energy of about 80%, indicating a significant loss in energy absorption capacity for longer web panels, as shown in Fig. 15a. Increasing the opening ratio $\:\mathrm{d}/{\mathrm{H}}_{\mathrm{a}\mathrm{v}\mathrm{g}}$ reduced the total strain energy by about 45–60%, showing that larger openings significantly limit post-buckling deformation capacity as shown in Fig. 15b. In comparison, the effect of tapering angle is relatively limited, as changing $\:{\uptheta\:}$ within the studied range causes about 10% variation in total strain energy on average as shown in Fig. 15c. Web slenderness had the strongest effect, with increasing $\:{\uplambda\:}$ ($\:{\mathrm{H}}_{\mathrm{a}\mathrm{v}\mathrm{g}}/{\mathrm{t}}_{\mathrm{w}})$ over the investigated range leads to a reduction of about 70% in total strain energy as presented in Fig. 15d. Overall, these trends indicate that total strain energy is mainly controlled by panel aspect ratio, opening ratio, and web slenderness, while the tapering angle plays a secondary role. Total strain energy is used as a performance indicator because it combines both load resistance and deformation capacity. Therefore, it reflects the ability of the girder panel to sustain post-buckling deformation and absorb energy before reaching its ultimate state.

## Development of design equation for ultimate shear capacity of tapered web panel of plate girders

This section reformulates the ultimate shear capacity model in a mechanics-based format compatible with the Eurocode 3 shear-resistance philosophy. The reference resistance is expressed using the steel shear-yield term, $\:{\mathrm{f}}_{\mathrm{y}}{\mathrm{A}}_{\mathrm{w}}/\sqrt{3}$, while the reduction due to web slenderness is introduced through a buckling-based factor. The effects of the web opening, panel aspect ratio, and tapering angle are then included through dimensionless modifiers calibrated against the verified FE database.

### Elastic shear buckling basis

For a slender web panel, the elastic critical shear stress may be written in terms of the shear buckling coefficient and the representative web depth:

1 $$\:{{\uptau\:}}_{\mathrm{c}\mathrm{r}}\:=\:{\mathrm{k}}_{{\uptau\:}}\:\frac{{{\uppi\:}}^{2}\mathrm{E}}{12(1-{{\upnu\:}}^{2})}\:{\left(\frac{{\mathrm{t}}_{\mathrm{w}}}{{\mathrm{H}}_{\mathrm{a}\mathrm{v}\mathrm{g}}}\right)}^{2}$$

where k_τ_ is the shear buckling coefficient, E is Young’s modulus, ν is Poisson’s ratio, t_w_ is the web thickness, and $H_{{avg}}$ is the average web depth of the tapered panel.

The shear buckling coefficient was expressed as:

2 $$\begin{array}{*{20}c} {k_{\tau } = 4 + \frac{{5.34}}{{\left( {B/H} \right)^{2} }}} & {if} & {(B/H) \le 1.0} \\ {k_{\tau } = 5.34 + \frac{4}{{\left( {B/H} \right)^{2} }}} & {if} & {(B/H)> 1.0} \\ \end{array}$$

where $\:\mathrm{B}$ is the web panel length and $\:\mathrm{H}$ is the larger web depth used in defining the panel aspect ratio.

### Slenderness-based reduction factor

The non-dimensional web slenderness is defined by comparing the yield stress with the elastic critical shear stress:

3 $$\:{{\uplambda\:}}_{\mathrm{w}}\:=\:\sqrt{\frac{{\mathrm{f}}_{\mathrm{y}}}{{\sqrt{3\:}\:.\:\:{\uptau\:}}_{\mathrm{c}\mathrm{r}}}}$$

where $\:{\mathrm{f}}_{\mathrm{y}}$is the steel yield stress.

The post-buckling shear resistance is represented through the following web reduction factor:

4 $$\:{{\upchi\:}}_{\mathrm{w}}\:=\:\frac{1}{\sqrt{1\:+\:{{{\uplambda\:}}_{\mathrm{w}}}^{\mathrm{n}}}}$$

The exponent n controls the sensitivity of the model to web slenderness and is obtained by nonlinear regression using the verified FE results.

### Geometric modifiers

The opening effect is expressed by combining the direct reduction in effective shear area with the nonlinear stress concentration around the opening:

5 $$\:{{\upeta\:}}_{\mathrm{o}}\:=\:(1\:-\:{{\upbeta\:}}_{1}\:\frac{\mathrm{d}}{{\mathrm{H}}_{\mathrm{a}\mathrm{v}\mathrm{g}}})(1\:-\:{{\upbeta\:}}_{2}\:{\left(\frac{\mathrm{d}}{{\mathrm{H}}_{\mathrm{a}\mathrm{v}\mathrm{g}}}\right)}^{2})$$

where $\:\mathrm{d}$ is the diameter of the circular opening, while $\:{{\upbeta\:}}_{1}$ and $\:{{\upbeta\:}}_{2}$ are calibrated factors. The first term accounts for the direct loss of web material due to the opening, while the second term reflects the nonlinear reduction associated with stress concentration around the opening.

The influence of panel aspect ratio is introduced through a power modifier reflecting the change in shear-buckling restraint with panel length:

6 $$\:{{\upeta\:}}_{\mathrm{a}}\:=\:{\left(\frac{\mathrm{H}}{\mathrm{B}}\right)}^{\mathrm{m}}$$

where $\:\mathrm{m}$ is a calibrated exponent. This term reflects the influence of the panel length relative to the web depth on the shear-buckling response.

The tapering angle modifies the shear-flow trajectories and is represented using a linear function of tan θ:

7 $$\:{{\upeta\:}}_{{\uptheta\:}}\:=\:1\:+\:{\upalpha\:}\:\mathrm{t}\mathrm{a}\mathrm{n}{\uptheta\:}$$

where $\:{\uptheta\:}$ is the tapering angle and $\:{\upalpha\:}$ is the calibrated factor. This term accounts for the observed influence of tapering on the shear-flow path and the post-buckling response of the web panel.

### Final proposed equation and exact regression calibration

Combining the EC3-aligned shear-yield base, the web slenderness reduction factor, and the geometric modifiers gives the proposed prediction equation:

8 $$\:{\mathrm{V}}_{\mathrm{p}\mathrm{r}\mathrm{e}\mathrm{d}}\:=\:\frac{{\mathrm{f}}_{\mathrm{y}}\:{\mathrm{A}}_{\mathrm{w}}}{\sqrt{3}\sqrt{1\:+\:{{{\uplambda\:}}_{\mathrm{w}}}^{\mathrm{n}}}}\:{{\upeta\:}}_{\mathrm{o}}\:{{\upeta\:}}_{\mathrm{a}}\:{{\upeta\:}}_{{\uptheta\:}}$$

where:

$$\:{\mathrm{A}}_{\mathrm{w}}\text{}={\mathrm{H}}_{\mathrm{a}\mathrm{v}\mathrm{g}}{\mathrm{t}}_{\mathrm{w}}$$

**Fig. 16:**
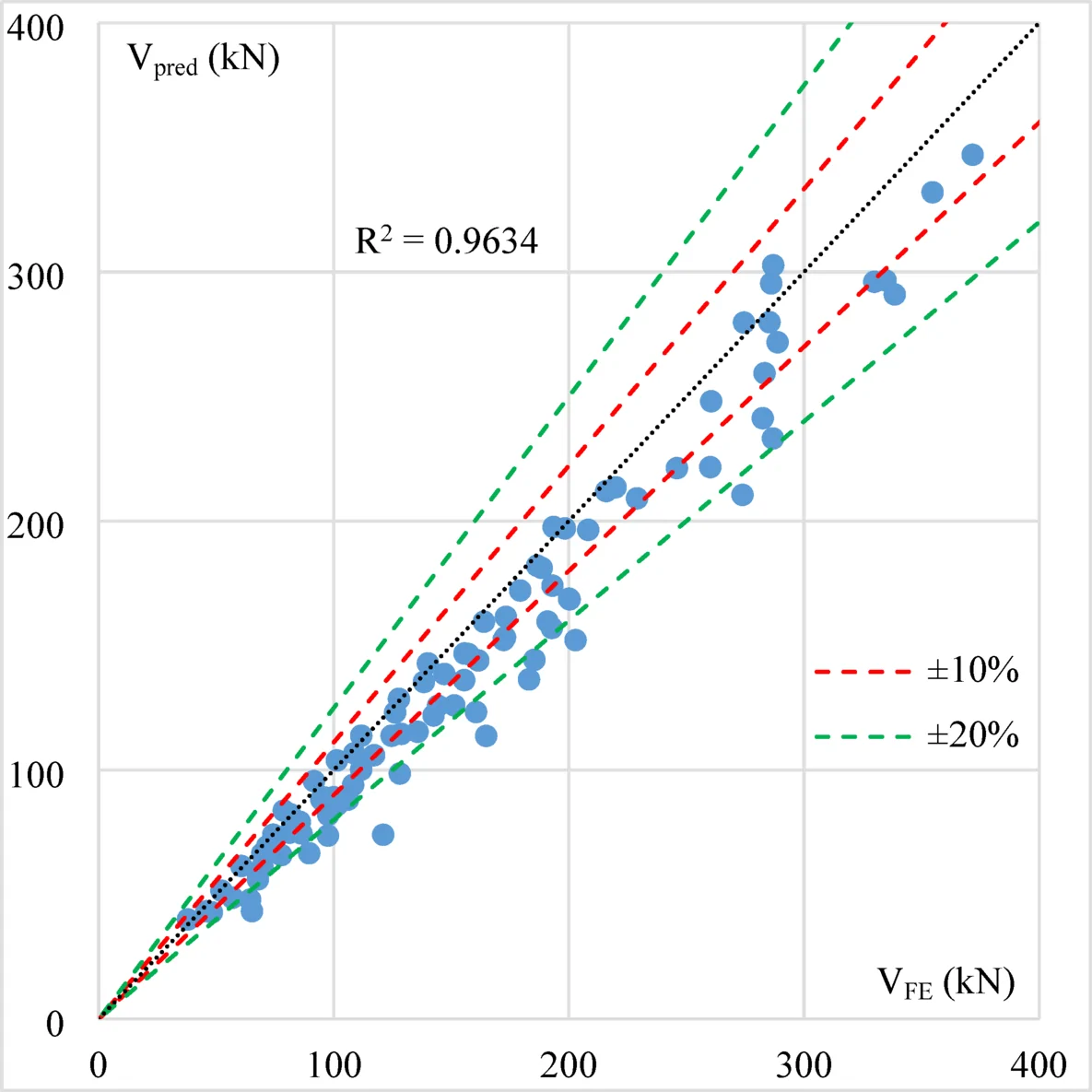
Comparison of proposed-model predictions with FE ultimate shear capacities.

The calibrated coefficients and statistical performance are summarized in Table 7. The calibrated model gives a mean prediction ratio, $\:{V}_{\mathrm{p}\mathrm{r}\mathrm{e}\mathrm{d}}/{V}_{\mathrm{F}\mathrm{E}}$, of 0.902, indicating a conservative prediction of the FE ultimate shear capacity on average. The standard deviation and coefficient of variation of the prediction ratio are 0.092 and 10.21%, respectively. The regression dataset comprised 90 solid-web and circular-opening specimens exhibiting the web-governed failure mode FM1. Of these specimens, 77 (85.56%) were predicted at or below the corresponding FE capacity, while 13 (14.44%) were overestimated, with a maximum overestimation of 6.34%. The seven square-opening specimens were excluded from the regression dataset because the proposed equation was calibrated specifically for solid-web and circular-opening specimens. Figure 16 compares the predictions of the proposed equation with the FE results. The proposed equation is applicable only to the web-governed ultimate shear capacity represented by FM1. Specimens governed by FM2 and FM3 were also excluded from the regression dataset.

**Table 7:** Calibrated coefficients and statistical performance of the proposed model.

Factor	Symbol	Value/range
Opening linear factor	β1	0.5939
Opening quadratic factor	β2	0.9266
Aspect-ratio exponent	m	0.0447
Tapering-angle factor	α	0.9165
Slenderness exponent	n	0.9637
Correlation coefficient	R	0.982
Coefficient of determination	R²	0.963
Root average squared error	RASE	13.43%
Mean prediction ratio	V_pred_/V_FE_	0.902
Standard deviation of V_pred_/V_FE_	SD (V_pred_/V_FE_)	0.092
Normalized standard deviation	NSD	0.937

*NSD is reported as the ratio between the standard deviation of predicted capacities and that of FE capacities. The calibration was performed using solid and circular-opening FE models only; square-opening cases were not used in the regression.

### Applicability and design use

The proposed model should be used only for the design of tapered plate girders with a circular web opening centred in the web panel and within the ranges listed in Table 8. Extrapolation beyond these limits is not recommended because the regression was calibrated using the verified FE database of the present study. For openings other than circular openings, the equation should be treated as indicative unless additional calibration data are available. Furthermore, the proposed model is limited to the web-governed failure mode FM1, characterized by local web buckling, stress concentration around the opening, and subsequent web yielding. Other governing mechanisms, such as compression-flange yielding or local buckling (FM2 and FM3), Vierendeel action around the web opening, or possible interaction between these mechanisms, are outside the scope of the present formulation and are recommended for further investigation.

**Table 8:** Applicability limit.

Parameter	Range
Panel aspect ratio (B/H)	0.67–1.50
Opening ratio (d/H_avg_)	0–0.67
Tapering angle (θ)	0°–25°
*Average web slenderness (H_avg_/t_w_)	78–300

*The given limit is for slender sections and the boarders of non-compact sections.

## Evaluation of proposed design model

The proposed EC3-aligned mechanics-based design model was evaluated against selected models available in the literature to assess its accuracy and consistency in predicting ultimate shear capacity. The comparison includes AISC 360^18^, AISC Design Guide 02^19^, AISC Design Guide 25^20^, Eurocode 3^21^, the Egyptian Code of Practice for Steel Construction and Bridges (ECP)^22^ and empirical models proposed by Serror et al.^2^, Ibrahim et al.^6^ and R. I. Shahin et al.^13^. The procedures used to compute ultimate shear capacity for each methodology are described in detail in Taher et al.^23^, and the same implementations are adopted here to ensure consistency.

For design methods that do not directly consider web openings, Their effect was represented using the net web area, which was determined by subtracting the opening area from the gross web area.

Table 9 compares the predictions of the considered design methods with the FE results using the correlation coefficient (R), normalized standard deviation (NSD), and root average squared error (RASE). The value of R indicates how closely each method follows the trend of the FE results. The normalized standard deviation (NSD) is the ratio of the standard deviation of the capacities predicted by each method to the standard deviation of the corresponding FE capacities. (RASE) reflects the overall prediction error. Therefore, better agreement is generally associated with a higher R, comparable scatter, and a lower RASE.

The proposed model showed the best agreement with the FE results, with high correlation, low scatter, and relatively small prediction errors. This indicates that the equation provides accurate predictions of the ultimate shear capacity within the investigated range while remaining simple enough for practical design.

**Table 9:** Comparison of the predictive performance of the evaluated design methodologies.

Rank	Methodology	Correlation coefficient (*R*)	NSD	RASE (%)
1	Proposed design model	0.982	0.937	13.4%
2	R. I. Shahin et al.^13^	0.921	0.913	39.8%
3	Eurocode 3^21^	0.913	0.902	41.0%
4	Ibrahim et al.^6^	0.915	0.830	41.2%
5	Serror et al.^2^	0.902	0.886	43.2%
6	DG-25^20^	0.913	1.201	50.0%
7	AISC 360^18^	0.904	1.171	50.4%
8	DG-02^19^	0.885	1.132	53.0%
9	ECP^22^	0.883	1.146	53.1%

## Conclusion

This study combined experimental verification with a finite element parametric database of 115 models to investigate the shear behaviour of web-tapered plate girders with web openings. The numerical results were then used to develop a practical design expression. Within the studied ranges, the following conclusions can be drawn:

- As the panel aspect ratio $\:\left(\mathrm{B}/\mathrm{H}\right)$ increased, it caused a clear reduction in performance. The ultimate shear capacity decreased by about 30–40%, while ductility decreased by approximately 60–70%. The total strain energy also dropped by nearly 80%. These results indicate that longer web panels lose both strength and post-buckling deformation capacity.
- Increasing the opening ratio (d/H_avg_) had the most detrimental effect. Compared with solid webs, specimens with large openings exhibited an average 60–70% reduction in ultimate shear capacity, 40–55% reduction in initial stiffness, 50–60% reduction in ductility, and 45–60% reduction in total strain energy, confirming that opening size governs both elastic and post-buckling response.
- The tapering angle (θ) showed a secondary influence within the investigated range (0°–25°). Ultimate shear capacity increased modestly by about 10–15%, while changes in initial stiffness, ductility, and total strain energy remained within about 10%, indicating that tapering mainly redistributes stresses rather than altering global response.
- Web slenderness (H_avg_/t_w_) strongly controlled strength and failure mode. Increasing slenderness caused a 70–75% reduction in ultimate shear capacity and about a 70% reduction in total strain energy, while ductility increased by 20–40% due to the earlier onset of buckling, which reduces the elastic displacement more rapidly than the ultimate displacement.
- Failure was web-governed in the majority of cases and was characterized by local web buckling that led to stress concentrations around the web opening. Non-web failures occurred mainly in stockier webs and selected combinations of panel aspect ratio, opening ratio, and tapering angle, where compression-flange yielding or local buckling governed.
- For geometrically matched specimens, circular openings consistently showed better performance than square openings, with increases in ultimate shear capacity ranging from 7% to 25%, and the difference becoming more pronounced as the opening ratio increased.
- An EC3-aligned mechanics-based design equation for ultimate shear capacity (V_u_) was developed using the circular-opening FE dataset. The proposed model incorporates web slenderness, opening ratio, panel aspect ratio, and tapering angle through physically interpretable modifiers, and reflects the combined influence of elastic shear buckling and post-buckling response. Comparison with selected literature methodologies showed that the calibrated equation provides the lowest RASE and a conservative mean prediction ratio of 0.902.

Within the investigated ranges, the proposed equation provides a simple, transparent, and mechanically interpretable tool for predicting the ultimate shear capacity of tapered web panels of plate girders with circular web openings.

## Data Availability

All data supporting the findings of this study are available within the paper in Table 3.
